# Biological networks in gestational diabetes mellitus: insights into the mechanism of crosstalk between long non-coding RNA and N^6^-methyladenine modification

**DOI:** 10.1186/s12884-022-04716-w

**Published:** 2022-05-03

**Authors:** Runyu Du, Yu Bai, Ling Li

**Affiliations:** Department of Endocrinology, Shengjing Hospital of China Medical University, No. 36, Sanhao Street, Heping District, Shenyang, 110004 Liaoning China

**Keywords:** Gestational diabetes mellitus, Mechanism, Long non-coding RNA, N^6^-methyladenine modification

## Abstract

**Background:**

Gestational diabetes mellitus (GDM) is one of the most common complications of pregnancy. The mechanism underlying the crosstalk between long non-coding RNAs (lncRNAs) and N^6^-methyladenine (m6A) modification in GDM remain unclear.

**Methods:**

We generated a lncRNA-mediated competitive endogenous RNA (ceRNA) network using comprehensive data from the Gene Expression Omnibus database, published data, and our preliminary findings. m6A-related lncRNAs were identified based on Pearson correlation coefficient (PCC) analysis using our previous profiles. An integrated pipeline was established to constructed a m6A-related subnetwork thereby predicting the potential effects of the m6A-related lncRNAs.

**Results:**

The ceRNA network was composed of 16 lncRNAs, 17 microRNAs, 184 mRNAs, and 338 edges. Analysis with the Kyoto Encyclopedia of Genes and Genomes database demonstrated that genes in the ceRNA network were primarily involved in the development and adverse outcomes of GDM, such as those in the fatty acid-metabolism pathway, the peroxisome proliferator-activated receptor signaling pathway, and thyroid hormone signaling pathway. Four m6A-related lncRNAs were involved in the ceRNA network, including *LINC00667*, *LINC01087*, *AP000350.6*, and *CARMN*. The m6A-related subnetwork was generated based on these four lncRNAs, their ceRNAs, and their related m6A regulators. Genes in the subnetwork were enriched in certain GDM-associated hormone (thyroid hormone and oxytocin) signaling pathways. *LINC00667* was positively correlated with an m6A “reader” (*YTHDF3*; PCC = 0.95) and exhibited the highest node degree in the ceRNA network. RIP assays showed that YTHDF3 directly bind *LINC00667*. We further found that *MYC* possessed the highest node degree in a protein–protein interaction network and competed with *LINC00667* for miR-33a-5p. qPCR analysis indicated that *LINC00667*, *YTHDF3* and *MYC* levels were upregulated in the GDM placentas, while miR-33a-5p was downregulated. In a support-vector machine classifier, an m6A-related module composed of *LINC00667*, *YTHDF3*, *MYC*, and miR-33a-5p showed excellent classifying power for GDM in both the training and the testing dataset, with an accuracy of 76.19 and 71.43%, respectively.

**Conclusions:**

Our results shed insights into the potential role of m6A-related lncRNAs in GDM and have implications in terms of novel therapeutic targets for GDM.

**Supplementary Information:**

The online version contains supplementary material available at 10.1186/s12884-022-04716-w.

## Background

Gestational diabetes mellitus (GDM), a condition involving metabolic dysfunction during pregnancy, can cause short-term and long-term adverse effects on both the mother and offspring [1]. It has been reported that GDM affects 2–5% of pregnancies worldwide, due to a diverse genetic background and epigenetic modifications that occur in response to nutritional and environmental factors [1, 2]. Currently, the understanding of the precise etiological mechanisms of GDM remains unclear.

Dysregulation of long non-coding lncRNAs (lncRNAs) has been reported to participate in numerous human diseases [3–5], and aberrant lncRNA expression is associated with GDM pathogenesis [6–9]. It has been reported that metastasis-associated lung adenocarcinoma transcript 1 (MALAT1) expression was elevated in GDM placentas, and its over-expression could suppress the proliferation, invasion, and migration of trophoblast cells via the TGF-β/NF-κB signaling pathway [7]. Reduced expression of lncRNA plasmacytoma variant translocation 1 (PVT1) was observed in the placentas of patients with GDM and preeclampsia, and knocking down PVT1 enhanced apoptosis and inhibited the proliferation, migration, and invasion of trophoblast cells through the PI3K/Akt pathway [6]. lncRNAs exhibit extensive functions and regulate the expression of genes at multiple levels. Several lncRNAs can function as competitive endogenous RNAs (ceRNAs) to regulate the expression of downstream genes by competing for their shared microRNAs (miRNAs) [10, 11]. Ye et al. [12] revealed that lncRNA maternally expressed gene 3 (MEG3) was up-regulated in the human umbilical vein endothelial cells (HUVECs) extracted from GDM pregnancies, and influence the fetal endothelial function through targeting AFF1 via sponging miR-370-3p. Overexpression of MEG3 was also obseved in the blood and placental villous tissue in pregnant GDM patients, and MEG3 could serve as a ceRNA of miR-345-3p to regulate the biological behavior and cell cycle of trophoblasts [9].

Chemical modifications occurring in lncRNAs can modify their secondary structure, splicing, degradation, or molecular stability, which can influence the expression of the lncRNAs and their downstream elements [13, 14]. N^6^-methyladenine RNA modification (m6A) is the most prevalent type of RNA epigenetic process and is regulated by m6A regulators, including methyltransferases (“writers”), signal transducers (“readers”), and demethylases (“erasers”) [13]. Recent evidence indicates that perturbations of m6A modifications dysregulate glucose/lipid metabolism and the immune/inflammatory response, thereby contributing to obesity, diabetes and cardiovascular diseases [15–17]. However, the knowledge regarding GDM is still in its infancy.

In this study, we focused on a lncRNA-mediated ceRNA network, m6A-related lncRNAs, crosstalk between them, and the potential effects on GDM.

## Materials and methods

### High-throughput expression profiles for GDM

Expression profiles for lncRNAs and mRNAs were generated by our research team [18] and are referred to here collectively as the GSE Shengjing profile. Samples were extracted from the placentas (maternal side) of women with normal glucose tolerance (NGT; *n* = 3) and GDM (n = 3). The human mRNA-expression datasets GSE2956, GSE19649, and GSE70493 were downloaded from the Gene Expression Omnibus database (https://www.ncbi.nlm.nih.gov/geo/). GSE2956 contains data for placental samples from 6 patients (GDM; *n* = 3 and NGT; n = 3). GSE19649 consists of data from one pooled GDM placental tissue and one pooled healthy placental tissue. The expression data in GSE70493 were obtained for validation and contain 63 maternal side placental samples (GDM; *n* = 32 and NGT; *n* = 31).

### Screening of differentially expressed RNAs

Using DEseq2 [19], we identified differentially expressed mRNAs (DEMs) and differentially expressed lncRNAs (DELs) in GSE Shengjing profile, based on the criteria of |log_2_ fold change| > 1 and adjusted *P* value < 0.05. A total of 172 lncRNAs and 142 mRNAs were differentially expressed in GSE Shengjing profile, respectively. Of these, 86 lncRNAs and 67 mRNAs were upregulated, and 86 lncRNAs and 75 mRNAs were downregulated. All these differentially expressed genes can also be identified using Limma package [20]. We only extracted lncRNAs with matching transcript IDs for analysis. Genes without official symbols were removed, and all symbols were converted to symbols approved by the HUGO Gene Nomenclature Committee. Heatmaps were conducted using the ‘pheatmap’ package of R software. The aberrantly expressed mRNAs in GSE2956 and GSE19649 that met the criterion of |log_2_ fold-change| > 1 were also pooled as DEMs for subsequent analysis.

The lncRNA-mediated ceRNA network was constructed on the basis that the miRNA was shared by any pair of ceRNA genes. In such cases, the reliability of miRNA was critical to establish a reliable lncRNA-miRNA-mRNA network. We systematically reviewed PubMed and pooled the dysregulated placenta-specific microRNAs (miRNAs) in GDM with ≥2 supporting experiments [21–27]. Those differentially expressed miRNAs (DEMis) with contradictory findings in different studies were further excluded.

### Collection of m6A regulators and identification of m6A-Related DELs

We pooled 21 m6A regulators from previous publications, including writers (*METTL14*, *METTL16*, *METTL3*, *RBM15*, *RBM15B*, *VIRMA* [*KIAA1429*], *WTAP*, and *ZC3H13*), readers (*HNRNPA2B1*, *HNRNPC*, *IGF2BP1*, *IGF2BP2*, *IGF2BP3*, *RBMX*, *YTHDC1*, *YTHDC2*, *YTHDF1*, *YTHDF2*, and *YTHDF3*), and erasers (*FTO* and *ALKBH5*). Pearson correlation coefficient (PCC) analysis between m6A regulators and DELs was conducted to identify the m6A-related DELs. A |PCC| of > 0.9 and *P* value of < 0.01 were used as the cutoff criteria.

### Construction of a lncRNA–miRNA–mRNA network associated with GDM

miRNA–lncRNA interactions were obtained from DIANA-LncBase v3 and contained > 500,000 experimentally supported miRNA targets on non-coding transcripts [28]. DIANA-TarBase [29] was used to retrieve the mRNA–miRNA-association data with experimental evidence. Finally, a lncRNA–miRNA–mRNA-regulatory network was constructed based on ceRNA theory using matching DEL–DEMi and DEMi–DEM pairs. Cytoscape 3.7.2 software (http://www.cytoscape.org/) was used to visualize the ceRNA network. All node degrees were calculated. Gene Ontology (GO) and Kyoto Encyclopedia of Genes and Genomes (KEGG) pathways were analyzed using the ‘enrichplot’ package of R software. A *P* value of < 0.05 was set as the cutoff. The Search Tool for the Retrieval of Interacting Genes [30] was applied to predict the protein-protein interactions (PPIs) of DEMs in the ceRNA network with a confidence of > 0.4. We visualized the PPI network using Cytoscape and excavated the most tightly interconnected modules using the MCODE plug-in. We also screened for hub genes in the PPI network using CytoHubb with a node degree of > 15.

### Identification of the m6A-related subnetwork and module

To filter the core m6A-related DELs, the m6A-related DELs were overlapped with lncRNAs in the ceRNA network. The m6A-Related subnetwork composed of the core m6A-related DELs, their ceRNAs, and the host m6A regulators was visualized by Cytoscape, and the enrichment assay was further analyzed. An m6A-related module containing a lncRNA with the highest node degree in the ceRNA network, the gene with the highest node degree in the PPI network, their shared miRNA target, and the host m6A gene of the lncRNA were extracted.

### Construction of a Support-Vector Machine (SVM) classifier

The support-vector machine (SVM) is comprised of a set of supervised-learning methods for performing binary and multi-class classifications, which has been used to classify and diagnose diseases [31–33]. A SVM classifier based on the selected m6A-related module was established to distinguish samples from subjects with GDM and healthy subjects by analyzing m6A-related module genes using the python package ‘scikit-learn 0.24.0’. Independent training/testing procedures using the SVM classifier were performed to predict the disease status associated with samples in GSE70493.

### Clinical samples and cell culture

All protocols for the use of human samples were approved by the Ethics Committee of Shengjing Hospital of China Medical University, in accordance with the Helsinki Guidelines. Thirty-two patients with GDM and 32 healthy controls were recruited from Shengjing Hospital of China Medical University. GDM was diagnosed according to the Chinese Current Care Guidelines for GDM [34]. The control group was matched to the GDM group through a 1:1 pattern according to age and pre-pregnancy body mass index (BMI). Patients who met the criteria as follows were excluded: younger than 18 years old, history of diabetes, infective or inflammatory diseases, hypertension, chronic diseases (thyroid dysfunction, cardio-cerebrovascular diseases, renal failure, etc.), multiple pregnancies, or used assistive reproductive technology. After cesarean section, placental tissues were collected and stored at − 80 °C. The maternal information and data on neonatal outcomes were collected from medical records. HTR-8/SVneo cells were cultured in RPMI-1640 (Gibco, Grand Island, NY, USA) supplemented with 10% fetal bovine serum (FBS, Hyclone, Logan, UT, USA) at 37 °C in 5% CO2.

### Quantitative real-time polymerase chain reaction (qPCR)

We used TRIzol reagent (Invitrogen, Carlsbad, CA, USA) to extract total RNA from tissues or cells. RNA purity and concentration were determined using a NANO 2000 Spectrophotometer (Thermo Fisher Scientific, Waltham, MA, USA) at 260 and 280 nm. cDNA was generated from RNA using the BioTeke super RT kit (Bioteke, Beijing, China) according to the manufacturer’s protocol. Subsequently, qPCR was carried out using a SYBR GREEN mastermix (Solarbio, Beijing, China). Data were analyzed using the 2^-ΔΔCt^ method and normalized to the expression levels of β-Actin or U6. All primers are described in Table S1.

### RNA immunoprecipitation (RIP) assay

RIP assay was performed using RIP Kit (Millipore, Billerica, MA, USA) according to the manufacturer’s instructions. Briefly, HTR-8/SVneo cells were lysed with RIP lysate buffer. The lysate was incubated overnight in RIP buffer with magnetic beads coupled to YTHDF3 or IgG antibodies (Abcam, Cambridge, UK) at 4 °C. The expression of *LINC00667* was analyzed by qPCR.

### Statistical analysis

The Shapiro test and Levene’s test were used to determine the normal distribution and homogeneity, respectively. Quantitative variables are presented as mean ± standard deviation, whereas qualitative variables are presented as counts and/or percentages. Associations between quantitative variables were assessed using the Student’s t-test or the Mann–Whitney U-test. Qualitative variables were compared using the Chi-square test. Graphing and statistical analyses were performed using GraphPad Prism 9, R software (version 4.0.3) and Python (version 3.8). *P* < 0.05 was considered statistically significant. All reactions in vitro were performed in triplicate.

## Results

### Filtering results for DELs, DEMis, and DEMs

**Fig. 1:**
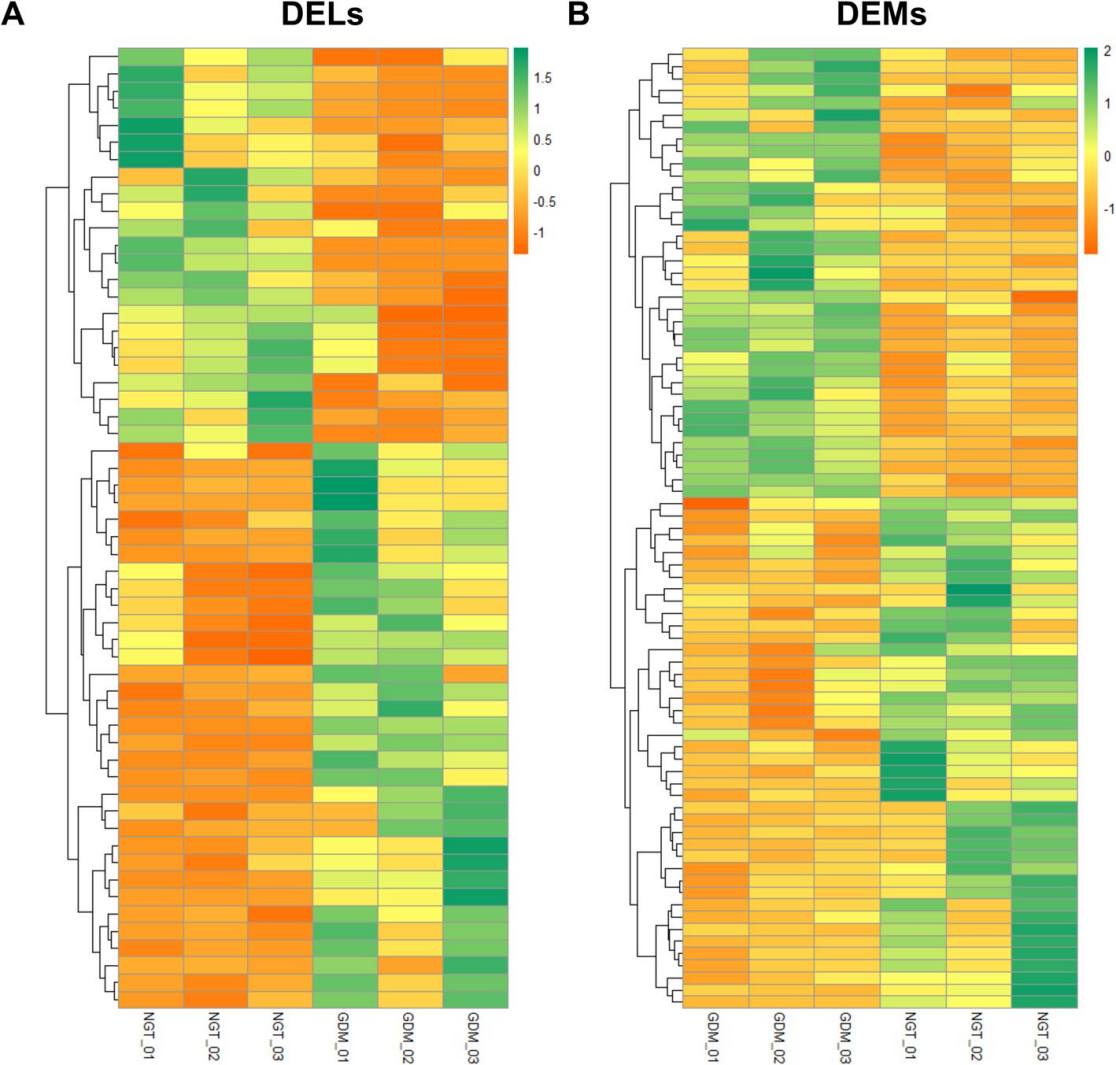
Hierarchical clustering of DELs and DEMs in the GSE Shengjing profile (**A**) The heatmap of the DELs. **B** The heatmap of the DEMs. The up- and down-regulation are indicated with orange and green color, respectively

Based on the criteria described above, we identified 56 DELs, 38 DEMis, and 540 DEMs. Of these, 33 lncRNAs, 22 miRNAs, and 283 mRNAs were upregulated, and 23 lncRNAs, 16 miRNAs, and 257 mRNAs were downregulated. The heatmaps of the DELs and DEMs found in GSE Shengjing profile are shown in Fig. 1. The details of the pooled DEMis and the information related to the DEMs are listed in Tables 1 and 2, respectively. The list of all DEMs identified in this study is shown in Table S2.

**Table 1:** Detailed information related to the pooled DEMis

Reference	DEMi name	Location	Trend
Gillet 2019 [26]	miR-122-5p	Placental exosomes	+
	miR-132-3p	Placental exosomes	+
	miR-1323	Placental exosomes	+
	miR-136-5p	Placental exosomes	+
	miR-182-3p	Placental exosomes	+
	miR-210-3p	Placental exosomes	+
	miR-29a-3p	Placental exosomes	+
	miR-29b-3p	Placental exosomes	+
	miR-342-3p	Placental exosomes	+
	miR-520 h	Placental exosomes	+
Ding 2018 [21]	miR-138-5p	Placenta	–
	miR-202-5p	Placenta	+
	miR-210-5p	Placenta	–
	miR-3158-5p	Placenta	–
	miR-4732-3p	Placenta	–
Nair 2018 [22]	miR-125a-3p	Placenta, placental-derived exosomes, circulating exosomes and skeletal muscle	+
	miR-99b-5p	Placenta, placental-derived exosomes, circulating exosomes and skeletal muscle	+
	miR-197-3p	Placenta, placental-derived exosomes, circulating exosomes and skeletal muscle	+
	miR-22-3p	Placenta, placental-derived exosomes, circulating exosomes and skeletal muscle	+
	miR-224-5p	Placenta, placental-derived exosomes, circulating exosomes and skeletal muscle	+
	miR-584-5p	Placenta, placental-derived exosomes	+
	miR-186-5p	Placenta, placental-derived exosomes	+
	miR-433-3p	Placenta, placental-derived exosomes	+
	miR-423-3p	Placenta, placental-derived exosomes	+
	miR-208a-3p	Placenta, placental-derived exosomes	–
	miR-335-5p	Placenta, placental-derived exosomes	–
	miR-451a	Placenta, placental-derived exosomes	–
	miR-145-3p	Placenta, placental-derived exosomes	–
	miR-369-3p	Placenta, placental-derived exosomes	–
	miR-483-3p	Placenta, placental-derived exosomes	–
	miR-203a-3b	Placenta, placental-derived exosomes	–
	miR-574-3p	Placenta, placental-derived exosomes	–
	miR-144-3p	Placenta, placental-derived exosomes	–
	miR-6795-5p	Placenta, placental-derived exosomes	–
	miR-550a-3-3p	Placenta, placental-derived exosomes	–
	miR-411-5p	Placenta, placental-derived exosomes	–
	miR-140-3p	Placenta, placental-derived exosomes	–
Li 2018 [27]	*MIR96*	Placenta	–
Xu 2017 [23]	miR-503	Placenta	+
Muralimanoharan 2016 [24]	miR-143	Placenta	–
Li 2015 [25]	miR-508-3p	Placenta	+
	miR-27a	Placenta	–
	miR-9	Placenta	–
	miR-92a	Placenta	–
	miR-30d	Placenta	–
	miR-362 -5p	Placenta	–
	miR-502-5p	Placenta	–
	miR-33a	Placenta	–

**Table 2:** Overall information related to the DEGs identified in this study

	Upregulated	Downregulated	Total
GSE19649	108	35	143
GSE2956	133	185	318
GSEShengjing	42	37	79

### Construction of the ceRNA network

**Fig. 2:**
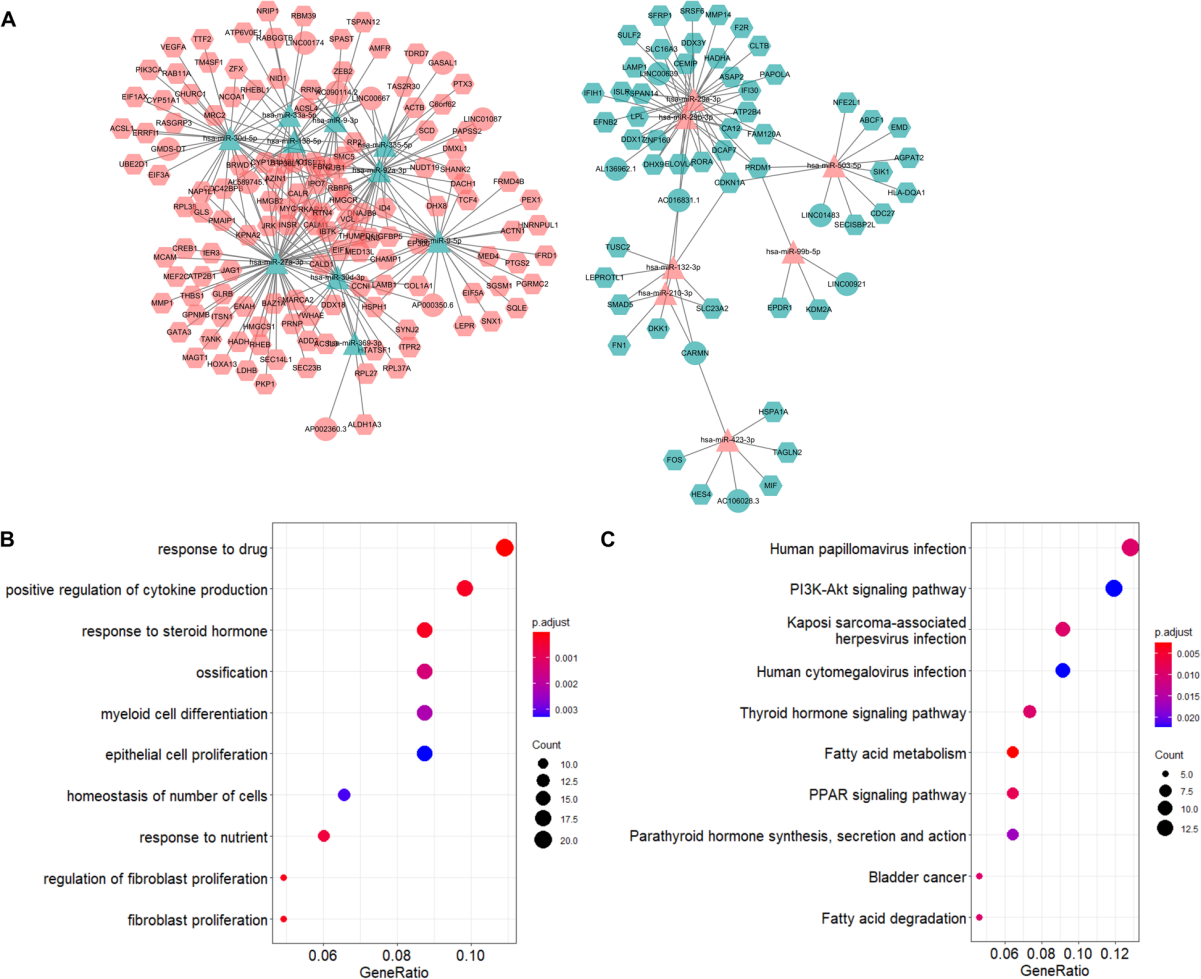
The lncRNA-mediated ceRNA network in GDM and functional analysis ranked according to the −log (*P* value). **A** Depiction of the lncRNA-mediated ceRNA network in GDM. The differentially expressed lncRNAs, mRNAs, and miRNAs are indicated with circle, hexagon, and triangle, respectively. The upregulation and downregulation are indicated with pink and blue shading, respectively. **B** The top 10 GO biological process categories. **C** The enriched KEGG pathways

**Fig. 3:**
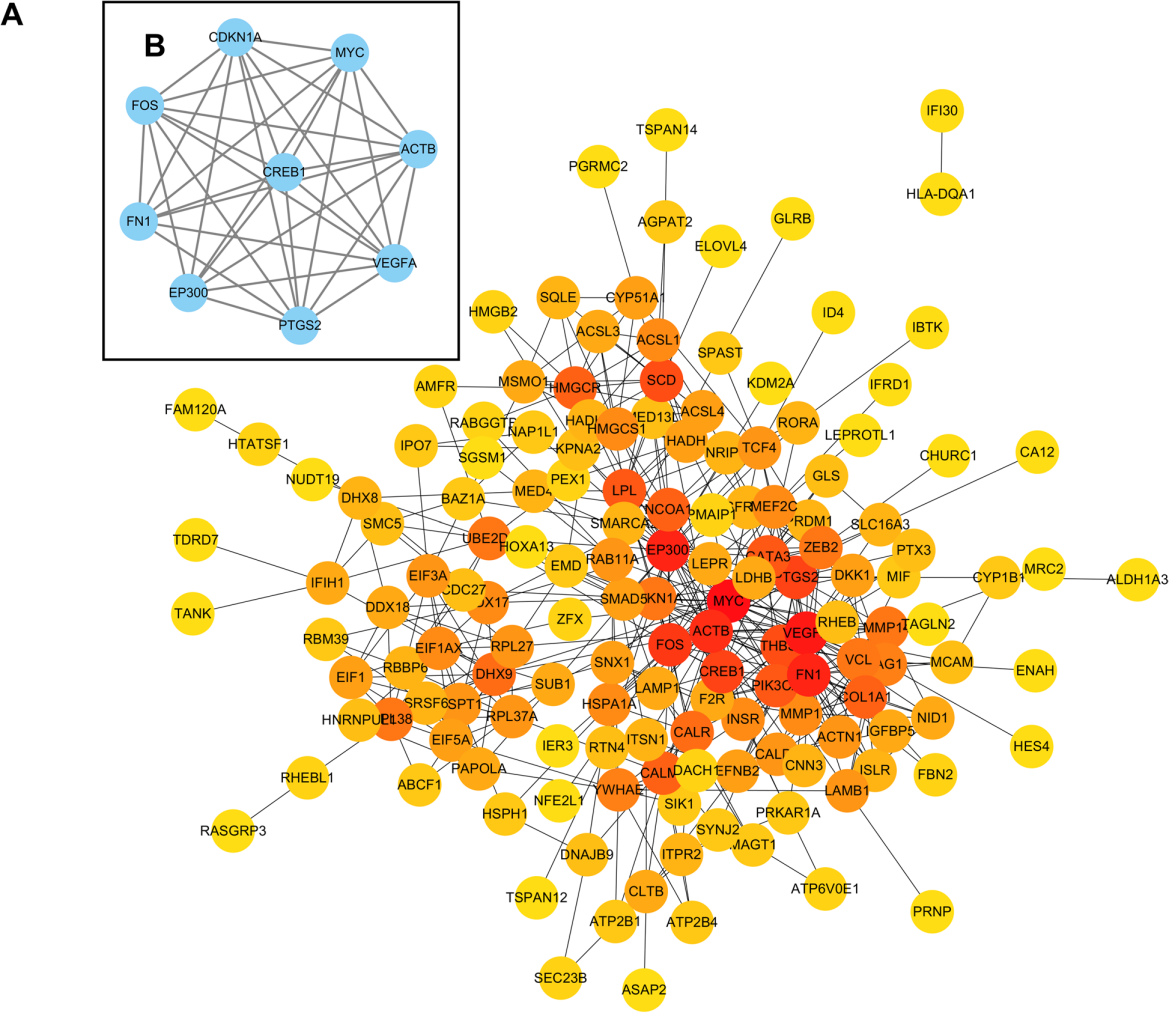
Construction of a PPI network. **A** PPI network of DEMs in the ceRNA network (colored according to the node degree). **B** The key module was identified using the MCODE plug-in

A lncRNA–miRNA–mRNA-regulatory network for GDM was constructed (Fig. 2A) and was found to consist of 16 lncRNA nodes, 17 miRNA nodes, 184 mRNA nodes, and 338 edges. The node degrees of the DELs in the ceRNA network are shown in Table 3. *LINC00667* was identified as the hub lncRNA as it exhibited the highest degree in the ceRNA network. Based on the GO category, biological process, DEMs in the ceRNA network mainly were enriched for response to drug, response to steroid hormone, fibroblast cell proliferation, positive regulation of cytokin production, and response to steroid hormone (Fig. 2B). KEGG pathways were mainly enriched in fatty acid metabolism, the peroxisome proliferator-activated receptor (PPAR) signaling pathway, and the thyroid hormone signaling pathway (Fig. 2C). A PPI network composed of 152 nodes and 475 edges was constructed and visualized (Fig. 3A). The key module (score = 8.75) was identified from the PPI network (Fig. 3B). The hub genes of the PPI network and the key module nodes are listed in Table 4.

**Table 3:** List of all DELs in the ceRNA network

DEL number	Name	Node degree	Trend
1	*LINC00667*	5	Upregulated
2	*AC016831.1*	4	Downregulated
3	*CARMN*	3	Downregulated
4	*AC090114.2*	3	Upregulated
5	*AL589745.1*	3	Upregulated
6	*LINC00639*	2	Downregulated
7	*AP000350.6*	2	Upregulated
8	*AL136962.1*	1	Downregulated
9	*AC106028.3*	1	Downregulated
10	*LINC01483*	1	Downregulated
11	*LINC00921*	1	Downregulated
12	*GMDS-DT*	1	Upregulated
13	*GASAL1*	1	Upregulated
14	*LINC00174*	1	Upregulated
15	*AP002360.3*	1	Upregulated
16	*LINC01087*	1	Upregulated

**Table 4:** Hub genes and key module nodes in the PPI network

Hub gene		Key module	
Annotation	Degree	Annotation	Degree
*MYC*	36	MYC	36
*VEGFA*	32	VEGFA	32
*FN1*	30	EP300	30
*EP300*	30	FN1	30
*ACTB*	28	ACTB	28
*FOS*	22	FOS	22
*CREB1*	16	PTGS2	16
*PTGS2*	16	CREB1	16
*THBS1*	15	CDKN1A	11
*SCD*	15		

### Identification of the m6A-related DELs in GDM

**Fig. 4:**
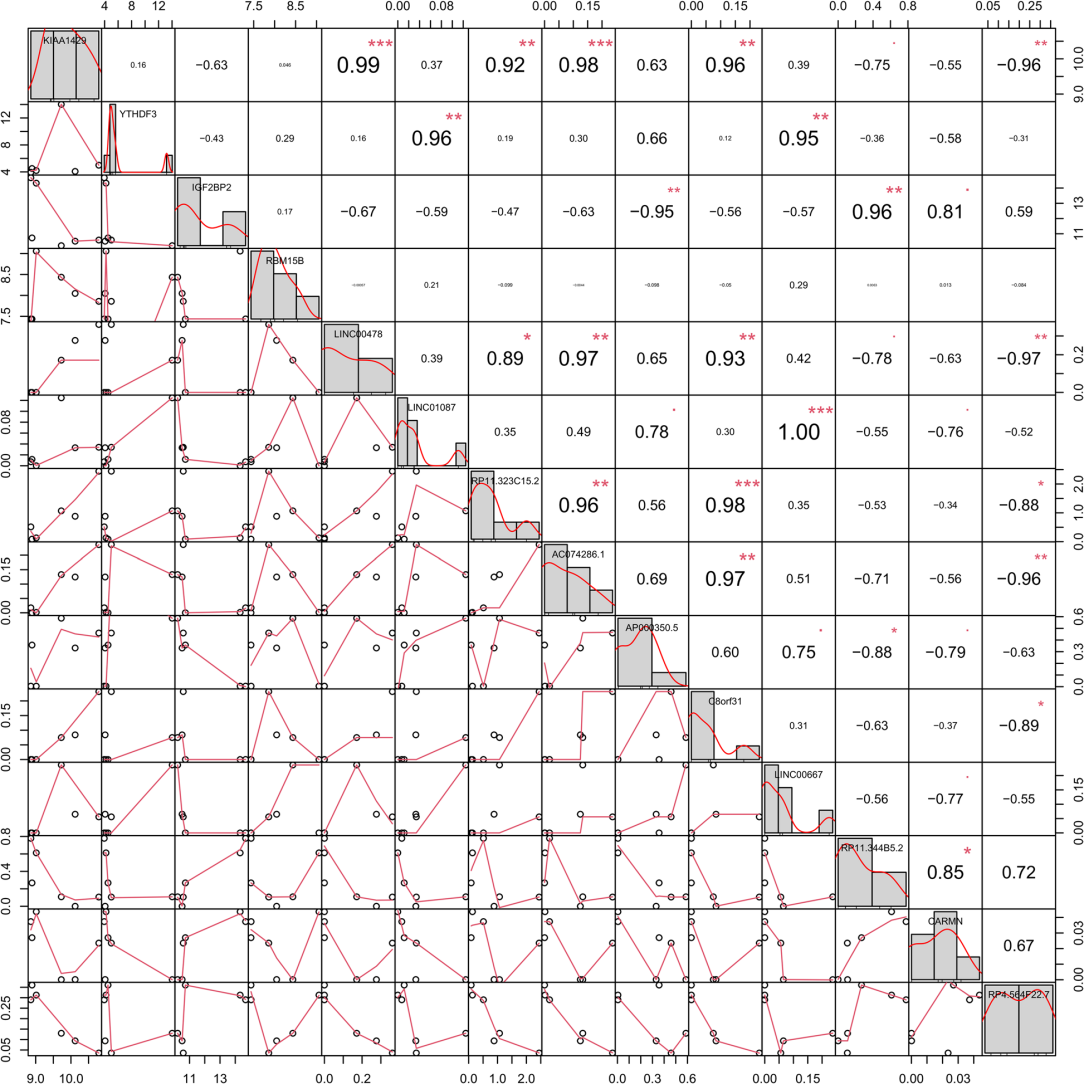
Correlation heatmap of DELs and m6A regulators

To identify m6A-related lncRNAs, we conducted Pearson correlation analysis using the expression matrixes of DELs and 21 m6A regulators from the GSE Shengjing profile. A PPC of > 0.9 and a *P* value of < 0.01 were set as the cutoff criteria. Ten DELs were significantly correlated with four m6A-related genes, as shown in Fig. 4.

### Characterization of the m6A-related subnetwork and module

**Fig. 5:**
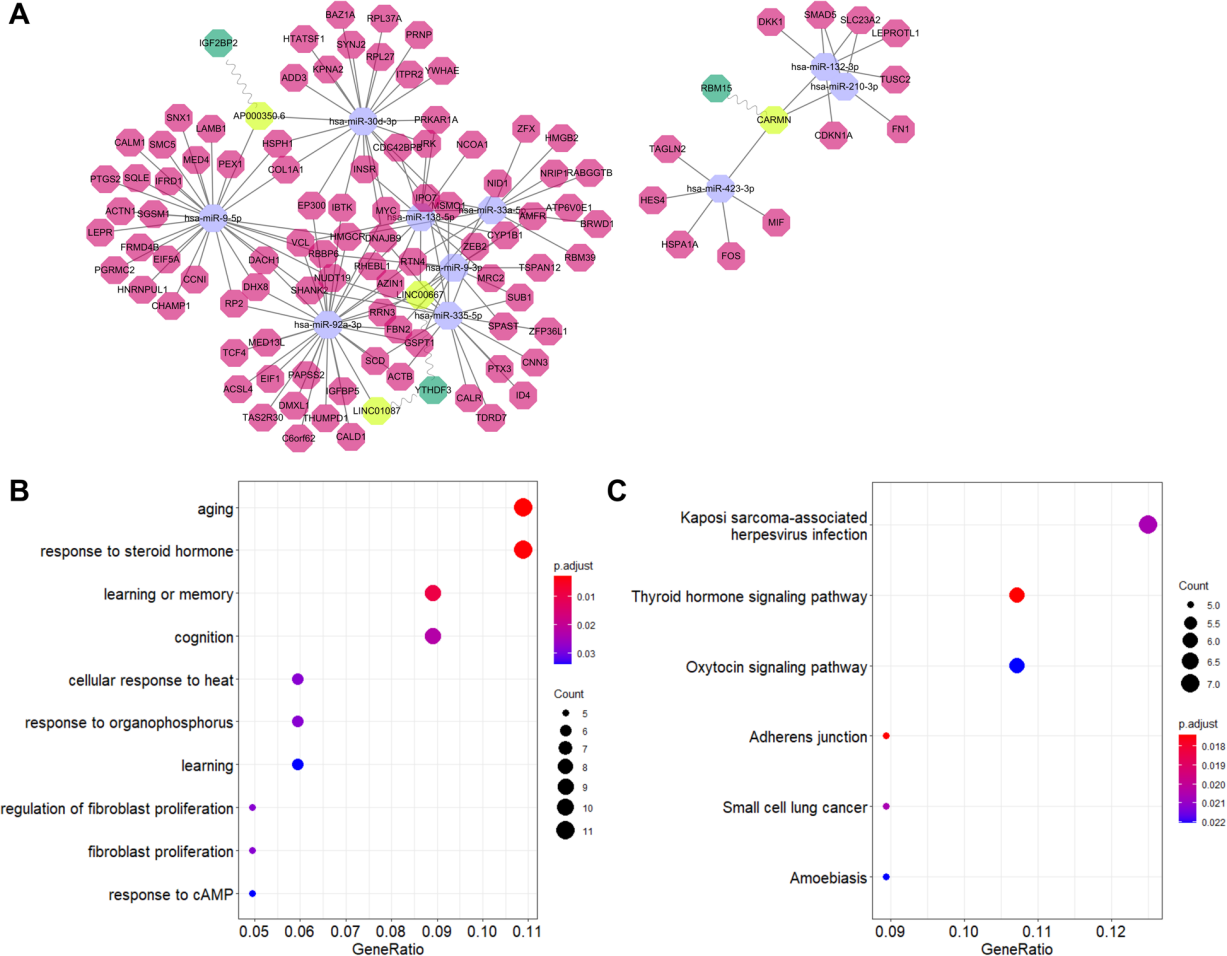
The m6A-related subnetwork in GDM and functional analysis ranked according to the −log (P value). **A** Depiction of the m6A-related subnetwork in GDM. The differentially expressed lncRNAs, mRNAs, miRNAs, and m6A regulators are indicated with yellow, pink, purple, and green shading, respectively. **B** The top 10 GO biological process categories. (C) The enriched KEGG pathways

Four m6A-related DELs were involved in the ceRNA network, including *LINC00667*, *LINC01087*, *AP000350.6*, and *CARMN*. A m6A-related subnetwork was identified containing 4 lncRNAs, 10 miRNAs, 101 mRNAs, and 3 m6A regulators (Fig. 5A). Functional analysis indicated that the major BP terms were aging, response to the steroid hormone, learning and cognition, and cell proliferation (Fig. 5B). KEGG pathways were mainly enriched in the thyroid hormone signaling pathway, Oxytocin signaling pathway, virus infection, and cancer (Fig. 5C).

**Fig. 6:**
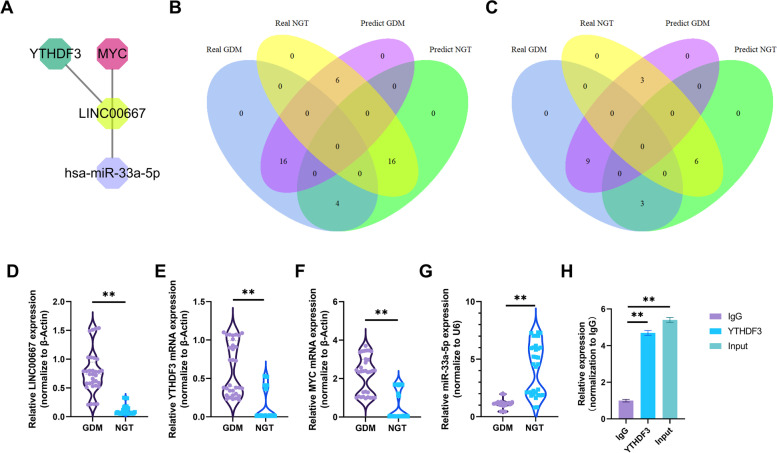
Identification of an m6A-related module. **A** The m6A-related module consisted of *LINC00667, YTHDF3, MYC*, and miR-33a-5p. **B** Venn diagram showing the overlap between modeled (predicted) groups and real groups in the training dataset. **C** Venn diagram showing the overlap between modeled (predicted) groups and real groups in the testing dataset. **D**-**G** The RNA expression levels of *LINC00667, YTHDF3, MYC*, and miR-33a-5p were analyzed using qPCR. ** *P* < 0.01. **H** The interaction between YTHDF3 and *LINC00667* was analyzed by RIP from HTR8/SVneo cells. ** *P* < 0.01

We further identified an m6A-related module to explore molecules associated with GDM, which consisted of four nodes (*LINC00667*, *YTHDF3, MYC*, and miR-33a-5p) (Fig. 6A). *LINC00667* exhibited the highest node degree among all lncRNAs in the ceRNA network. The heatmap (Fig. 4) shows that the expression level of the hub lncRNA (*LINC00667*) correlated with that of *YTHDF3* (PCC = 0.95), which is an m6A-related reader. MYC exhibited the highest node degree in the PPI network. The subnetwork plot (Fig. 5A) shows that *LINC00667* can act as ceRNA for miR-33a-5p to regulate *MYC*.

### Validation of the m6A-related module

The expression matrix of the four genes in the m6A-related module was extracted from dataset GSE92772, and then the SVM classifier was constructed. We found that 32 of 42 samples and 15 of 21 samples were correctly classified in training set and testing set, with an accuracy of 76.19 and 71.43%, respectively (Fig. 6B, C). We further determined the expression levels of *LINC00667*, *YTHDF3*, *MYC*, and miR-33a-5p in placental tissues (tissues of 32 GDM patients and 32 controls) by qPCR. The clinical data are summarized in Table S3. In addition to indicators of impaired glucose metabolism, we observed more weight gain during gestation on patients with GDM compared with control subjects. *LINC00667*, *YTHDF3*, *MYC* levels were significantly higher in the GDM group than in the NGT group (Fig. 6D-F), while miR-33a-5p was significantly decreased (Fig. 6G). A total of 47 m6A sites in *LINC00667* were predicted using RMBase [35]. The details of predicted m6A sites in *LINC00667* were shown in Supplementary file 1. Moreover, RIP assays showed that YTHDF3, which is a m6A reader, directly bind *LINC00667* (Fig. 6H)*.* These indicated that the stability of LINC00667 may be affected through m6A modification via YTHDF3*.* Existing literature indicates that both *LINC00667* and *MYC* serve as targets of miR-33a-5p [36–41]. This suggests that *YTHDF3/LINC00667/*miR-33a-5p/*MYC* axis maybe potential targets for research on the mechanism of GDM.

## Discussion

GDM is a condition of pregnancy-related hyperglycemia with increased morbidity and mortality, for both the mother and fetus. The etiology of GDM, which involves the genetic background and epigenetic modifications, remains unclear. We selected the placenta as the subject of this study based on its specific location between the maternal and fetal bloodstreams. The placenta is exposed to intrauterine conditions that adversely affect placental and fetal development, which explains why most GDM-related adverse pregnancy outcomes originate in the placenta [42].

As a recently discovered type of non-coding RNAs, lncRNAs participate in numerous cellular functions and metabolic diseases [43]. However, only a limited number of studies have been conducted to explore the relationship between lncRNAs and GDM. Cao et al. [44] performed a microarray-based expression-profile analysis, which revealed aberrantly expressed lncRNAs in umbilical cord blood exosomes from patients with GDM. *MEG3*, *MEG8*, and *MALAT1* upregulation, and *PVT1* downregulation were involved in GDM development and the adverse effects on offspring, which provides novel biomarkers or therapeutic targets for GDM [6, 7, 9, 45]. Certain lncRNAs, miRNAs, and genes can form ceRNA motifs, which may participate in the molecular mechanism of GDM. In this study, a lncRNA-mediated ceRNA regulatory network was generated and analyzed. Enrichment analysis was conducted to determine the biological functions enriched for among DEMs in the ceRNA network. Notably, several pathways were closely correlated with the development and the adverse outcomes of GDM, where the fatty acid-metabolism pathway, the PPAR signaling pathway, and the thyroid hormone signaling pathway were the top three pathways [46–49].

As the most abundant epigenetic form of mRNA and non-coding RNA (ncRNA) methylation, m6A modification plays vital roles in various diseases [15, 17, 50]. However, it remains unknown how m6A modification acts in a lncRNA-dependent manner in GDM. We identified m6A-related lncRNAs based on a dataset generated in our previous study [18]. The m6A-related lncRNAs were intersected with the DELs in the ceRNA network to obtain four lncRNAs: *LINC00667*, *LINC01087*, *AP000350.6*, and *CARMN*. Subsequently, a m6A-related subnetwork was generated based on these four lncRNAs, their ceRNAs, and their related m6A regulators. Genes in the subnetwork were enriched in certain hormone signaling pathways, including thyroid hormone and oxytocin (OT). Recent evidence indicates that perturbations of the thyroid hormone signaling pathway and antibodies are associated with GDM development and mal-outcome [49, 51]. Emerging evidence suggests a role of OT in the pathogenesis of insulin resistance, obesity, and dyslipidemia [52, 53], which are hallmarks or risk factors for GDM. A significant weight loss and improvement of insulin sensitivity, pancreatic β-cell responsivity, and lipid metabolism can be observed in rodents, nonhuman primates, and humans after chronic subcutaneous or intranasal OT treatment [54, 55]. *LINC00667* exhibited the highest node degree in the ceRNA network. Moreover, the genes in the *LINC00667*-mediated ceRNA subnetwork were functionally related to GDM by participating in the thyroid hormone signaling pathway. Consequently, *LINC00667* may notably contribute to the development of GDM. A total of 47 m6A modification sites in *LINC00667* were predicted using RMBase v2.0 [35]. Pearson correlation analysis suggested that *LINC00667* was positively correlated with *YTHDF3* (PCC = 0.95), an m6A “reader”. Moreover, RIP assays showed that YTHDF3 directly bind *LINC00667* in HTR8/SVneo cells. We further found that MYC possessed the highest node degree in the PPI network. Existing literature indicates that both *LINC00667* and *MYC* serve as targets of miR-33a-5p [36–41]. Through qPCR validation in 64 placental samples, we demonstrated that *LINC00667*, *YTHDF3*, *MYC* levels were significantly higher in the GDM group than in the NGT, while miR-33a-5p was significantly decreased. Furthermore, a SVM classifier for GDM was applied based on a module composed of *LINC00667*, *YTHDF3*, *MYC*, and miR-33a-5p. The expression matrix of these four genes was extracted based on data from 63 samples. Through the training and testing, this module showed good classifying power for GDM. Therefore, our findings suggested that this m6A-related module can be regarded as containing pivotal targets for research on the mechanism of GDM.

This study had a few limitations. A comprehensive analysis of the placenta and peripheral blood is warranted to verify the RNA expression, protein expression and m6A-modification status of the hub genes. The diagnostic ability of this m6A-related module may require further validation using a larger sample size. For subsequent research, more experiments in vitro regarding the biological functions of *YTHDF3/LINC00667/*miR-33a-5p/*MYC* axis should be incorporated to shed light into the mechanism of GDM.

## Conclusion

In conclusion, this study was focused on potential m6A-related lncRNAs and crosstalk with the lncRNA-mediated ceRNA network that affects GDM. Based on bioinformatics analysis, we identified an m6A-related module consisting of *LINC00667*, *YTHDF3*, *MYC*, and miR-33a-5p, which was used successfully to classify GDM and NGT samples. A comprehensive analysis of the placenta and peripheral blood using a larger sample size, combined with validation of intermolecular interactions in vivo and in vitro, is warranted to validate the accuracy and reliability of our findings in the future.

## Supplementary Information

**Additional file 1: Table S1**. Primer sequences.

**Additional file 2: Table S2**. List of all differentially expressed mRNAs (DEMs).

**Additional file 3: Table S3**. Clinical characteristics of patients included in the study.

**Additional file 4.** Supplementary file 1.

## Data Availability

The RNA profiles of datasets GSE2956, GSE19649, and GSE70493 were downloaded from the Gene Expression Omnibus database (https://www.ncbi.nlm.nih.gov/geo/). The miRNA-lncRNA interactions and mRNA-miRNA-association data were obtained from DIANA-LncBase v3 (http://www.microrna.gr/LncBase) and DIANA-TarBase (http://www.microrna.gr/tarbase), respectively. The m6A sites in LINC00667 were predicted using RMBase (http://rna.sysu.edu.cn/rmbase/).

## Abbreviations

ceRNA: competitive endogenous RNA
DEL: Differentially expressed lncRNA
DEM: Differentially expressed mRNA
DEMi: Differentially expressed miRNA
GDM: Gestational diabetes mellitus
GO: Gene Ontology
KEGG: Kyoto Encyclopedia of Genes and Genomes
lncRNA: long non-coding RNA
m6A: N^6^-methyladenine
miRNA: microRNA; NGT, normal glucose tolerance
PCC: Pearson correlation coefficient
PPI: Protein–protein interaction
SVM: Support-vector machine

## References

[1] Reece EA, Leguizamon G, Wiznitzer A (2009). Gestational diabetes: the need for a common ground. Lancet..

[2] American Diabetes A (2020). 2. Classification and diagnosis of diabetes: standards of medical Care in Diabetes-2020. Diabetes Care.

[3] Tsagakis I, Douka K, Birds I, Aspden JL (2020). Long non-coding RNAs in development and disease: conservation to mechanisms. J Pathol.

[4] Sanchez Calle A, Kawamura Y, Yamamoto Y, Takeshita F, Ochiya T (2018). Emerging roles of long non-coding RNA in cancer. Cancer Sci.

[5] Gupta SC, Awasthee N, Rai V, Chava S, Gunda V, Challagundla KB (2020). Long non-coding RNAs and nuclear factor-kappaB crosstalk in cancer and other human diseases. Biochim Biophys Acta Rev Cancer.

[6] Wang Q, Lu X, Li C, Zhang W, Lv Y, Wang L (2019). Down-regulated long non-coding RNA PVT1 contributes to gestational diabetes mellitus and preeclampsia via regulation of human trophoblast cells. Biomed Pharmacother.

[7] Zhang Y, Qu L, Ni H, Wang Y, Li L, Yang X (2020). Expression and function of lncRNA MALAT1 in gestational diabetes mellitus. Adv Clin Exp Med.

[8] Zhang Y, Wu H, Wang F, Ye M, Zhu H, Bu S (2018). Long non-coding RNA MALAT1 expression in patients with gestational diabetes mellitus. Int J Gynaecol Obstet.

[9] Zhang H (2019). Mechanism associated with aberrant lncRNA MEG3 expression in gestational diabetes mellitus. Exp Ther Med.

[10] Feng K, Liu Y, Xu LJ, Zhao LF, Jia CW, Xu MY (2018). Long noncoding RNA PVT1 enhances the viability and invasion of papillary thyroid carcinoma cells by functioning as ceRNA of microRNA-30a through mediating expression of insulin like growth factor 1 receptor. Biomed Pharmacother.

[11] Wu Y, Jia K, Wu H, Sang A, Wang L, Shi L (2019). A comprehensive competitive endogenous RNA network pinpoints key molecules in diabetic retinopathy. Mol Med Rep.

[12] Ye HH, Yang SH, Zhang Y (2018). MEG3 damages fetal endothelial function induced by gestational diabetes mellitus via AKT pathway. Eur Rev Med Pharmacol Sci.

[13] He RZ, Jiang J, Luo DX (2020). The functions of N6-methyladenosine modification in lncRNAs. Genes Dis.

[14] Lan Y, Liu B, Guo H (2021). The role of M(6) a modification in the regulation of tumor-related lncRNAs. Mol Ther Nucleic Acids.

[15] Zhong H, Tang HF, Kai Y (2020). N6-methyladenine RNA modification (m(6)a): an emerging regulator of metabolic diseases. Curr Drug Targets.

[16] Yang Y, Shen F, Huang W, Qin S, Huang JT, Sergi C (2019). Glucose is involved in the dynamic regulation of m6A in patients with type 2 diabetes. J Clin Endocrinol Metab.

[17] Berulava T, Buchholz E, Elerdashvili V, Pena T, Islam MR, Lbik D (2020). Changes in m6A RNA methylation contribute to heart failure progression by modulating translation. Eur J Heart Fail.

[18] Tang L, Li P, Li L (2020). Whole transcriptome expression profiles in placenta samples from women with gestational diabetes mellitus. J Diabetes Investig.

[19] Anders S, Huber W (2010). Differential expression analysis for sequence count data. Genome Biol.

[20] Ritchie ME, Phipson B, Wu D, Hu Y, Law CW, Shi W (2015). Limma powers differential expression analyses for RNA-sequencing and microarray studies. Nucleic Acids Res.

[21] Ding R, Guo F, Zhang Y, Liu XM, Xiang YQ, Zhang C (2018). Integrated Transcriptome sequencing analysis reveals role of miR-138-5p/ TBL1X in placenta from gestational diabetes mellitus. Cell Physiol Biochem.

[22] Nair S, Jayabalan N, Guanzon D, Palma C, Scholz-Romero K, Elfeky O (2018). Human placental exosomes in gestational diabetes mellitus carry a specific set of miRNAs associated with skeletal muscle insulin sensitivity. Clin Sci (Lond).

[23] Xu K, Bian D, Hao L, Huang F, Xu M, Qin J (2017). microRNA-503 contribute to pancreatic beta cell dysfunction by targeting the mTOR pathway in gestational diabetes mellitus. EXCLI J.

[24] Muralimanoharan S, Maloyan A, Myatt L (2016). Mitochondrial function and glucose metabolism in the placenta with gestational diabetes mellitus: role of miR-143. Clin Sci (Lond)..

[25] Li J, Song L, Zhou L, Wu J, Sheng C, Chen H (2015). A MicroRNA signature in gestational diabetes mellitus associated with risk of Macrosomia. Cell Physiol Biochem.

[26] Gillet V, Ouellet A, Stepanov Y, Rodosthenous RS, Croft EK, Brennan K (2019). miRNA profiles in extracellular vesicles from serum early in pregnancies complicated by gestational diabetes mellitus. J Clin Endocrinol Metab.

[27] Li L, Wang S, Li H, Wan J, Zhou Q, Zhou Y (2018). microRNA-96 protects pancreatic beta-cell function by targeting PAK1 in gestational diabetes mellitus. Biofactors..

[28] Karagkouni D, Paraskevopoulou MD, Tastsoglou S, Skoufos G, Karavangeli A, Pierros V (2020). DIANA-LncBase v3: indexing experimentally supported miRNA targets on non-coding transcripts. Nucleic Acids Res.

[29] Vlachos IS, Paraskevopoulou MD, Karagkouni D, Georgakilas G, Vergoulis T, Kanellos I (2015). DIANA-TarBase v7.0: indexing more than half a million experimentally supported miRNA:mRNA interactions. Nucleic Acids Res.

[30] Szklarczyk D, Gable AL, Lyon D, Junge A, Wyder S, Huerta-Cepas J (2019). STRING v11: protein-protein association networks with increased coverage, supporting functional discovery in genome-wide experimental datasets. Nucleic Acids Res.

[31] Peker M (2016). A decision support system to improve medical diagnosis using a combination of k-medoids clustering based attribute weighting and SVM. J Med Syst.

[32] Su J, Zhang Y, Su H, Zhang C, Li W (2017). A recurrence model for laryngeal cancer based on SVM and gene function clustering. Acta Otolaryngol.

[33] Winters-Hilt S, Merat S (2007). SVM clustering. BMC Bioinformatics.

[34] Jia W, Weng J, Zhu D, Ji L, Lu J, Zhou Z (2019). Standards of medical care for type 2 diabetes in China 2019. Diabetes Metab Res Rev.

[35] Xuan JJ, Sun WJ, Lin PH, Zhou KR, Liu S, Zheng LL (2018). RMBase v2.0: deciphering the map of RNA modifications from epitranscriptome sequencing data. Nucleic Acids Res.

[36] Boudreau RL, Jiang P, Gilmore BL, Spengler RM, Tirabassi R, Nelson JA (2014). Transcriptome-wide discovery of microRNA binding sites in human brain. Neuron..

[37] Gillen AE, Yamamoto TM, Kline E, Hesselberth JR, Kabos P (2016). Improvements to the HITS-CLIP protocol eliminate widespread mispriming artifacts. BMC Genomics.

[38] Hamilton MP, Rajapakshe KI, Bader DA, Cerne JZ, Smith EA, Coarfa C (2016). The landscape of microRNA targeting in prostate Cancer defined by AGO-PAR-CLIP. Neoplasia..

[39] Takeshita H, Shiozaki A, Bai XH, Iitaka D, Kim H, Yang BB (2013). XB130, a new adaptor protein, regulates expression of tumor suppressive microRNAs in cancer cells. PLoS One.

[40] Gottwein E, Corcoran DL, Mukherjee N, Skalsky RL, Hafner M, Nusbaum JD (2011). Viral microRNA targetome of KSHV-infected primary effusion lymphoma cell lines. Cell Host Microbe.

[41] Braun J, Misiak D, Busch B, Krohn K, Huttelmaier S (2014). Rapid identification of regulatory microRNAs by miTRAP (miRNA trapping by RNA in vitro affinity purification). Nucleic Acids Res.

[42] Brett KE, Ferraro ZM, Yockell-Lelievre J, Gruslin A, Adamo KB (2014). Maternal-fetal nutrient transport in pregnancy pathologies: the role of the placenta. Int J Mol Sci.

[43] Quinn JJ, Chang HY (2016). Unique features of long non-coding RNA biogenesis and function. Nat Rev Genet.

[44] Cao M, Zhang L, Lin Y, Li Z, Xu J, Shi Z (2020). Differential mRNA and long noncoding RNA expression profiles in umbilical cord blood Exosomes from gestational diabetes mellitus patients. DNA Cell Biol.

[45] Zhang W, Cao D, Wang Y, Ren W (2021). LncRNA MEG8 is upregulated in gestational diabetes mellitus (GDM) and predicted kidney injury. J Diabetes Complicat.

[46] Ogundipe E, Samuelson S, Crawford MA (2020). Gestational diabetes mellitus prediction? A unique fatty acid profile study. Nutr Diabetes.

[47] Nguyen-Ngo C, Jayabalan N, Salomon C, Lappas M (2019). Molecular pathways disrupted by gestational diabetes mellitus. J Mol Endocrinol.

[48] Hutter S, Knabl J, Andergassen U, Jeschke U (2013). The role of PPARs in placental immunology: a systematic review of the literature. PPAR Res.

[49] Sert UY, Buyuk GN, Engin Ustun Y, Ozgu Erdinc AS (2020). Is there any relationship between thyroid function abnormalities, thyroid antibodies and development of gestational diabetes mellitus (GDM) in pregnant women?. Medeni Med J.

[50] Chen J, Du B (2019). Novel positioning from obesity to cancer: FTO, an m(6) a RNA demethylase, regulates tumour progression. J Cancer Res Clin Oncol.

[51] Knabl J, de Maiziere L, Huttenbrenner R, Hutter S, Juckstock J, Mahner S (2020). Cell type- and sex-specific Dysregulation of thyroid hormone receptors in placentas in gestational diabetes mellitus. Int J Mol Sci.

[52] McCormack SE, Blevins JE, Lawson EA (2020). Metabolic effects of oxytocin. Endocr Rev.

[53] Iovino M, Messana T, Tortora A, Giusti C, Lisco G, Giagulli VA (2021). Oxytocin signaling pathway: from cell biology to clinical implications. Endocr Metab Immune Disord Drug Targets.

[54] Blevins JE, Baskin DG (2015). Translational and therapeutic potential of oxytocin as an anti-obesity strategy: insights from rodents, nonhuman primates and humans. Physiol Behav.

[55] Mohan S, McCloskey AG, McKillop AM, Flatt PR, Irwin N, Moffett RC (2020). Development and characterisation of novel, enzymatically stable oxytocin analogues with beneficial antidiabetic effects in high fat fed mice. Biochim Biophys Acta Gen Subj.

