# Supplementary material for: Biological networks in gestational diabetes mellitus: insights into the mechanism of crosstalk between long non-coding RNA and N6-methyladenine modification
Source: BMC Pregnancy Childbirth. 2022 May 3;22:384. doi: 10.1186/s12884-022-04716-w (PMC9066898; doi:10.1186/s12884-022-04716-w)
Supplement: Supplementary file 1 — Additional file 1: Table S1. Primer sequences. [file 12884_2022_4716_MOESM1_ESM.doc]

**Table S1. Primer sequences**

| **Gene** | **Forward** | **Reverse** |
| --- | --- | --- |
| ***LINC00667*** | ACACCTGCAATCCCAGCTAC | GTCTCGCTCCATCATCCAGG |
| ***YTHDF3*** | TCAGAGTAACAGCTATCCACCA | GGTTGTCAGATATGGCATAGGCT |
| **miR-33a-5p** | GTGCATTGTAGTTGCATTGCA | GTGCAGGGTCCGAGGTATTC |
| ***MYC*** | GGCTCCTGGCAAAAGGTCA | CTGCGTAGTTGTGCTGATGT |
| **β-actin** | CATGTACGTTGCTATCCAGGC | CTCCTTAATGTCACGCACGAT |
| **U6** | CTCGCTTCGGCAGCACA | AACGCTTCACGAATTTGCGT |
