# Supplementary material for: Biological networks in gestational diabetes mellitus: insights into the mechanism of crosstalk between long non-coding RNA and N6-methyladenine modification
Source: BMC Pregnancy Childbirth. 2022 May 3;22:384. doi: 10.1186/s12884-022-04716-w (PMC9066898; doi:10.1186/s12884-022-04716-w)
Supplement: Supplementary file 2 — Additional file 2: Table S2. List of all differentially expressed mRNAs (DEMs). [file 12884_2022_4716_MOESM2_ESM.docx]

**Table S2.** List of all differentially expressed mRNAs (DEMs).

| **Symbol** | **Description** | **HGNC ID** | **Location** | **Dataset** | **Direction** |
| --- | --- | --- | --- | --- | --- |
| *ANO3* | anoctamin 3 | HGNC:14004 | 11p14.2 | GSE19649 | up |
| *DEFA1* | defensin alpha 1 | HGNC:2761 | 8p23.1 | GSE19649 | up |
| *DEFA3* | defensin alpha 3 | HGNC:2762 | 8p23.1 | GSE19649 | up |
| *ATP5MK* | ATP synthase membrane subunit k | HGNC:30889 | 10q24.33 | GSE19649 | up |
| *MMP12* | matrix metallopeptidase 12 | HGNC:7158 | 11q22.2 | GSE19649 | up |
| *FCGBP* | Fc fragment of IgG binding protein | HGNC:13572 | 19q13.2 | GSE19649 | up |
| *FCGR3B* | Fc fragment of IgG receptor IIIb | HGNC:3620 | 1q23.3 | GSE19649 | up |
| *SGSM1* | small G protein signaling modulator 1 | HGNC:29410 | 22q11.23 | GSE19649 | up |
| *SCGN* | secretagogin, EF-hand calcium binding protein | HGNC:16941 | 6p22.2 | GSE19649 | up |
| *PTGS2* | prostaglandin-endoperoxide synthase 2 | HGNC:9605 | 1q31.1 | GSE19649 | up |
| *AHSP* | alpha hemoglobin stabilizing protein | HGNC:18075 | 16p11.2 | GSE19649 | up |
| *TNMD* | tenomodulin | HGNC:17757 | Xq22.1 | GSE19649 | up |
| *GABRB1* | gamma-aminobutyric acid type A receptor subunit beta1 | HGNC:4081 | 4p12 | GSE19649 | up |
| *PTX3* | pentraxin 3 | HGNC:9692 | 3q25.32 | GSE19649 | up |
| *TM4SF18* | transmembrane 4 L six family member 18 | HGNC:25181 | 3q25.1 | GSE19649 | up |
| *AFF2* | AF4/FMR2 family member 2 | HGNC:3776 | Xq28 | GSE19649 | up |
| *UST* | uronyl 2-sulfotransferase | HGNC:17223 | 6q25.1 | GSE19649 | up |
| *COL8A1* | collagen type VIII alpha 1 chain | HGNC:2215 | 3q12.1 | GSE19649 | up |
| *EDN1* | endothelin 1 | HGNC:3176 | 6p24.1 | GSE19649 | up |
| *CAMP* | cathelicidin antimicrobial peptide | HGNC:1472 | 3p21.31 | GSE19649 | up |
| *CHURC1* | churchill domain containing 1 | HGNC:20099 | 14q23.3 | GSE19649 | up |
| *DACH1* | dachshund family transcription factor 1 | HGNC:2663 | 13q21.33 | GSE19649 | up |
| *BBOX1* | gamma-butyrobetaine hydroxylase 1 | HGNC:964 | 11p14.2 | GSE19649 | up |
| *VNN2* | vanin 2 | HGNC:12706 | 6q23.2 | GSE19649 | up |
| *CCK* | cholecystokinin | HGNC:1569 | 3p22.1 | GSE19649 | up |
| *CETP* | cholesteryl ester transfer protein | HGNC:1869 | 16q13 | GSE19649 | up |
| *TMEM100* | transmembrane protein 100 | HGNC:25607 | 17q22 | GSE19649 | up |
| *HOXA13* | homeobox A13 | HGNC:5102 | 7p15.2 | GSE19649 | up |
| *RASGRP3* | RAS guanyl releasing protein 3 | HGNC:14545 | 2p22.3 | GSE19649 | up |
| *NNAT* | neuronatin | HGNC:7860 | 20q11.23 | GSE19649 | up |
| *CPXM2* | carboxypeptidase X, M14 family member 2 | HGNC:26977 | 10q26.13 | GSE19649 | up |
| *FCN3* | ficolin 3 | HGNC:3625 | 1p36.11 | GSE19649 | up |
| *HECW2* | HECT, C2 and WW domain containing E3 ubiquitin protein ligase 2 | HGNC:29853 | 2q32.3 | GSE19649 | up |
| *MEOX2* | mesenchyme homeobox 2 | HGNC:7014 | 7p21.2 | GSE19649 | up |
| *CD300LG* | CD300 molecule like family member g | HGNC:30455 | 17q21.31 | GSE19649 | up |
| *ZFX* | zinc finger protein X-linked | HGNC:12869 | Xp22.11 | GSE2956 | up |
| *ZFP36L1* | ZFP36 ring finger protein like 1 | HGNC:1107 | 14q24.1 | GSE2956 | up |
| *ZEB2* | zinc finger E-box binding homeobox 2 | HGNC:14881 | 2q22.3 | GSE2956 | up |
| *YWHAE* | tyrosine 3-monooxygenase/tryptophan 5-monooxygenase activation protein epsilon | HGNC:12851 | 17p13.3 | GSE2956 | up |
| *LYVE1* | lymphatic vessel endothelial hyaluronan receptor 1 | HGNC:14687 | 11p15.4 | GSE2956 | up |
| *VEGFA* | vascular endothelial growth factor A | HGNC:12680 | 6p21.1 | GSE2956 | up |
| *VCL* | vinculin | HGNC:12665 | 10q22.2 | GSE2956 | up |
| *UPF3A* | UPF3A regulator of nonsense mediated mRNA decay | HGNC:20332 | 13q34 | GSE2956 | up |
| *UBE2D1* | ubiquitin conjugating enzyme E2 D1 | HGNC:12474 | 10q21.1 | GSE2956 | up |
| *TUBGCP3* | tubulin gamma complex associated protein 3 | HGNC:18598 | 13q34 | GSE2956 | up |
| *TTF2* | transcription termination factor 2 | HGNC:12398 | 1p13.1 | GSE2956 | up |
| *TRIM5* | tripartite motif containing 5 | HGNC:16276 | 11p15.4 | GSE2956 | up |
| *TM4SF1* | transmembrane 4 L six family member 1 | HGNC:11853 | 3q25.1 | GSE2956 | up |
| *THBS1* | thrombospondin 1 | HGNC:11785 | 15q14 | GSE2956 | up |
| *THBD* | thrombomodulin | HGNC:11784 | 20p11.21 | GSE2956 | up |
| *TGFB1I1* | transforming growth factor beta 1 induced transcript 1 | HGNC:11767 | 16p11.2 | GSE2956 | up |
| *TERF1* | telomeric repeat binding factor 1 | HGNC:11728 | 8q21.11 | GSE2956 | up |
| *ZEB1* | zinc finger E-box binding homeobox 1 | HGNC:11642 | 10p11.22 | GSE2956 | up |
| *TCF4* | transcription factor 4 | HGNC:11634 | 18q21.2 | GSE2956 | up |
| *TCF7L2* | transcription factor 7 like 2 | HGNC:11641 | 10q25.2-q25.3 | GSE2956 | up |
| *TANK* | TRAF family member associated NFKB activator | HGNC:11562 | 2q24.2 | GSE2956 | up |
| *TAGLN* | transgelin | HGNC:11553 | 11q23.3 | GSE2956 | up |
| *SYNJ2* | synaptojanin 2 | HGNC:11504 | 6q25.3 | GSE2956 | up |
| *STXBP3* | syntaxin binding protein 3 | HGNC:11446 | 1p13.3 | GSE2956 | up |
| *HSPA13* | heat shock protein family A (Hsp70) member 13 | HGNC:11375 | 21q11.2 | GSE2956 | up |
| *SSB* | small RNA binding exonuclease protection factor La | HGNC:11316 | 2q31.1 | GSE2956 | up |
| *SQLE* | squalene epoxidase | HGNC:11279 | 8q24.13 | GSE2956 | up |
| *SPAST* | spastin | HGNC:11233 | 2p22.3 | GSE2956 | up |
| *SORBS1* | sorbin and SH3 domain containing 1 | HGNC:14565 | 10q24.1 | GSE2956 | up |
| *SNX1* | sorting nexin 1 | HGNC:11172 | 15q22.31 | GSE2956 | up |
| *SMARCA4* | SWI/SNF related, matrix associated, actin dependent regulator of chromatin, subfamily a, member 4 | HGNC:11100 | 19p13.2 | GSE2956 | up |
| *SMARCA2* | SWI/SNF related, matrix associated, actin dependent regulator of chromatin, subfamily a, member 2 | HGNC:11098 | 9p24.3 | GSE2956 | up |
| *SLC7A8* | solute carrier family 7 member 8 | HGNC:11066 | 14q11.2 | GSE2956 | up |
| *SLC22A3* | solute carrier family 22 member 3 | HGNC:10967 | 6q25.3 | GSE2956 | up |
| *SLC16A4* | solute carrier family 16 member 4 | HGNC:10925 | 1p13.3 | GSE2956 | up |
| *SHANK2* | SH3 and multiple ankyrin repeat domains 2 | HGNC:14295 | 11q13.3-q13.4 | GSE2956 | up |
| *SH3BP5* | SH3 domain binding protein 5 | HGNC:10827 | 3p25.1 | GSE2956 | up |
| *SRSF3* | serine and arginine rich splicing factor 3 | HGNC:10785 | 6p21.31-p21.2 | GSE2956 | up |
| *SEC24D* | SEC24 homolog D, COPII coat complex component | HGNC:10706 | 4q26 | GSE2956 | up |
| *SEC23B* | SEC23 homolog B, COPII coat complex component | HGNC:10702 | 20p11.23 | GSE2956 | up |
| *SEC22B* | SEC22 homolog B, vesicle trafficking protein | HGNC:10700 | 1p12 | GSE2956 | up |
| *SEC14L1* | SEC14 like lipid binding 1 | HGNC:10698 | 17q25.2-q25.3 | GSE2956 | up |
| *SCD* | stearoyl-CoA desaturase | HGNC:10571 | 10q24.31 | GSE2956 | up |
| *MSMO1* | methylsterol monooxygenase 1 | HGNC:10545 | 4q32.3 | GSE2956 | up |
| *S100A8* | S100 calcium binding protein A8 | HGNC:10498 | 1q21.3 | GSE2956 | up |
| *RTN4* | reticulon 4 | HGNC:14085 | 2p16.1 | GSE2956 | up |
| *RPS26* | ribosomal protein S26 | HGNC:10414 | 12q13.2 | GSE2956 | up |
| *RPS11* | ribosomal protein S11 | HGNC:10384 | 19q13.3 | GSE2956 | up |
| *RPS10* | ribosomal protein S10 | HGNC:10383 | 6p21.31 | GSE2956 | up |
| *RPL38* | ribosomal protein L38 | HGNC:10349 | 17q25.1 | GSE2956 | up |
| *RPL37A* | ribosomal protein L37a | HGNC:10348 | 2q35 | GSE2956 | up |
| *RPL27A* | ribosomal protein L27a | HGNC:10329 | 11p15.4 | GSE2956 | up |
| *RPL27* | ribosomal protein L27 | HGNC:10328 | 17q21.31 | GSE2956 | up |
| *RP2* | RP2 activator of ARL3 GTPase | HGNC:10274 | Xp11.3 | GSE2956 | up |
| *RBM39* | RNA binding motif protein 39 | HGNC:15923 | 20q11.22 | GSE2956 | up |
| *RHEB* | Ras homolog, mTORC1 binding | HGNC:10011 | 7q36.1 | GSE2956 | up |
| *RBBP6* | RB binding protein 6, ubiquitin ligase | HGNC:9889 | 16p12.1 | GSE2956 | up |
| *IPO7* | importin 7 | HGNC:9852 | 11p15.4 | GSE2956 | up |
| *RABGGTB* | Rab geranylgeranyltransferase subunit beta | HGNC:9796 | 1p31.1 | GSE2956 | up |
| *RAB11A* | RAB11A, member RAS oncogene family | HGNC:9760 | 15q22.31 | GSE2956 | up |
| *PTN* | pleiotrophin | HGNC:9630 | 7q33 | GSE2956 | up |
| *PRNP* | prion protein | HGNC:9449 | 20p13 | GSE2956 | up |
| *PRKAR1A* | protein kinase cAMP-dependent type I regulatory subunit alpha | HGNC:9388 | 17q24.2 | GSE2956 | up |
| *SRGN* | serglycin | HGNC:9361 | 10q22.1 | GSE2956 | up |
| *PMAIP1* | phorbol-12-myristate-13-acetate-induced protein 1 | HGNC:9108 | 18q21.32 | GSE2956 | up |
| *PLA2G5* | phospholipase A2 group V | HGNC:9038 | 1p36.13 | GSE2956 | up |
| *PLA2G2A* | phospholipase A2 group IIA | HGNC:9031 | 1p36.13 | GSE2956 | up |
| *PIK3CA* | phosphatidylinositol-4,5-bisphosphate 3-kinase catalytic subunit alpha | HGNC:8975 | 3q26.32 | GSE2956 | up |
| *PGRMC2* | progesterone receptor membrane component 2 | HGNC:16089 | 4q28.2 | GSE2956 | up |
| *PEX1* | peroxisomal biogenesis factor 1 | HGNC:8850 | 7q21.2 | GSE2956 | up |
| *PCTP* | phosphatidylcholine transfer protein | HGNC:8752 | 17q22 | GSE2956 | up |
| *PAPSS2* | 3'-phosphoadenosine 5'-phosphosulfate synthase 2 | HGNC:8604 | 10q23.2-q23.31 | GSE2956 | up |
| *AZIN1* | antizyme inhibitor 1 | HGNC:16432 | 8q22.3 | GSE2956 | up |
| *NRIP1* | nuclear receptor interacting protein 1 | HGNC:8001 | 21q11.2-q21.1 | GSE2956 | up |
| *NPAS2* | neuronal PAS domain protein 2 | HGNC:7895 | 2q11.2 | GSE2956 | up |
| *NID1* | nidogen 1 | HGNC:7821 | 1q42.3 | GSE2956 | up |
| *NCOA1* | nuclear receptor coactivator 1 | HGNC:7668 | 2p23.3 | GSE2956 | up |
| *NAP1L1* | nucleosome assembly protein 1 like 1 | HGNC:7637 | 12q21.1 | GSE2956 | up |
| *MYL9* | myosin light chain 9 | HGNC:15754 | 20q11.23 | GSE2956 | up |
| *MYC* | MYC proto-oncogene, bHLH transcription factor | HGNC:7553 | 8q24.21 | GSE2956 | up |
| *MRPL19* | mitochondrial ribosomal protein L19 | HGNC:14052 | 2p12 | GSE2956 | up |
| *MMRN1* | multimerin 1 | HGNC:7178 | 4q22.1 | GSE2956 | up |
| *MMP1* | matrix metallopeptidase 1 | HGNC:7155 | 11q22.2 | GSE2956 | up |
| *MEF2C* | myocyte enhancer factor 2C | HGNC:6996 | 5q14.3 | GSE2956 | up |
| *MCAM* | melanoma cell adhesion molecule | HGNC:6934 | 11q23.3 | GSE2956 | up |
| *LYN* | LYN proto-oncogene, Src family tyrosine kinase | HGNC:6735 | 8q12.1 | GSE2956 | up |
| *LEPR* | leptin receptor | HGNC:6554 | 1p31.3 | GSE2956 | up |
| *LEP* | leptin | HGNC:6553 | 7q32.1 | GSE2956 | up |
| *LDHB* | lactate dehydrogenase B | HGNC:6541 | 12p12.1 | GSE2956 | up |
| *LAMB1* | laminin subunit beta 1 | HGNC:6486 | 7q31.1 | GSE2956 | up |
| *KTN1* | kinectin 1 | HGNC:6467 | 14q22.1 | GSE2956 | up |
| *KPNA2* | karyopherin subunit alpha 2 | HGNC:6395 | 17q24.2 | GSE2956 | up |
| *KLF5* | Kruppel like factor 5 | HGNC:6349 | 13q22.1 | GSE2956 | up |
| *CEMIP* | cell migration inducing hyaluronidase 1 | HGNC:29213 | 15q25.1 | GSE2956 | up |
| *JAG1* | jagged canonical Notch ligand 1 | HGNC:6188 | 20p12.2 | GSE2956 | up |
| *ITSN1* | intersectin 1 | HGNC:6183 | 21q22.11 | GSE2956 | up |
| *ITPR2* | inositol 1,4,5-trisphosphate receptor type 2 | HGNC:6181 | 12p11.23 | GSE2956 | up |
| *ITGB5* | integrin subunit beta 5 | HGNC:6160 | 3q21.2 | GSE2956 | up |
| *INSR* | insulin receptor | HGNC:6091 | 19p13.2 | GSE2956 | up |
| *CXCR2* | C-X-C motif chemokine receptor 2 | HGNC:6027 | 2q35 | GSE2956 | up |
| *IL1RL1* | interleukin 1 receptor like 1 | HGNC:5998 | 2q12.1 | GSE2956 | up |
| *IGFBP5* | insulin like growth factor binding protein 5 | HGNC:5474 | 2q35 | GSE2956 | up |
| *IGF2* | insulin like growth factor 2 | HGNC:5466 | 11p15.5 | GSE2956 | up |
| *ID4* | inhibitor of DNA binding 4, HLH protein | HGNC:5363 | 6p22.3 | GSE2956 | up |
| *IBTK* | inhibitor of Bruton tyrosine kinase | HGNC:17853 | 6q14.1 | GSE2956 | up |
| *HTATSF1* | HIV-1 Tat specific factor 1 | HGNC:5276 | Xq26.3 | GSE2956 | up |
| *HSPA1A* | heat shock protein family A (Hsp70) member 1A | HGNC:5232 | 6p21.33 | GSE2956 | up |
| *HMGCS1* | 3-hydroxy-3-methylglutaryl-CoA synthase 1 | HGNC:5007 | 5p12 | GSE2956 | up |
| *HMGCR* | 3-hydroxy-3-methylglutaryl-CoA reductase | HGNC:5006 | 5q13.3 | GSE2956 | up |
| *HMGB2* | high mobility group box 2 | HGNC:5000 | 4q34.1 | GSE2956 | up |
| *HLA-DPA1* | major histocompatibility complex, class II, DP alpha 1 | HGNC:4938 | 6p21.32 | GSE2956 | up |
| *HADH* | hydroxyacyl-CoA dehydrogenase | HGNC:4799 | 4q25 | GSE2956 | up |
| *H2BC8* | H2B clustered histone 8 | HGNC:4746 | 6p22.2 | GSE2956 | up |
| *GSPT1* | G1 to S phase transition 1 | HGNC:4621 | 16p13.13 | GSE2956 | up |
| *GPNMB* | glycoprotein nmb | HGNC:4462 | 7p15.3 | GSE2956 | up |
| *GNGT1* | G protein subunit gamma transducin 1 | HGNC:4411 | 7q21.3 | GSE2956 | up |
| *GLS* | glutaminase | HGNC:4331 | 2q32.2 | GSE2956 | up |
| *GHR* | growth hormone receptor | HGNC:4263 | 5p13.1-p12 | GSE2956 | up |
| *GBP1* | guanylate binding protein 1 | HGNC:4182 | 1p22.2 | GSE2956 | up |
| *GATA3* | GATA binding protein 3 | HGNC:4172 | 10p14 | GSE2956 | up |
| *GALNT11* | polypeptide N-acetylgalactosaminyltransferase 11 | HGNC:19875 | 7q36.1 | GSE2956 | up |
| *FLT1* | fms related receptor tyrosine kinase 1 | HGNC:3763 | 13q12.3 | GSE2956 | up |
| *FLI1* | Fli-1 proto-oncogene, ETS transcription factor | HGNC:3749 | 11q24.3 | GSE2956 | up |
| *FKBP1A* | FKBP prolyl isomerase 1A | HGNC:3711 | 20p13 | GSE2956 | up |
| *FBN2* | fibrillin 2 | HGNC:3604 | 5q23.3 | GSE2956 | up |
| *FBLN1* | fibulin 1 | HGNC:3600 | 22q13.31 | GSE2956 | up |
| *ACSL4* | acyl-CoA synthetase long chain family member 4 | HGNC:3571 | Xq23 | GSE2956 | up |
| *ACSL3* | acyl-CoA synthetase long chain family member 3 | HGNC:3570 | 2q36.1 | GSE2956 | up |
| *ACSL1* | acyl-CoA synthetase long chain family member 1 | HGNC:3569 | 4q35.1 | GSE2956 | up |
| *FABP5* | fatty acid binding protein 5 | HGNC:3560 | 8q21.13 | GSE2956 | up |
| *FABP4* | fatty acid binding protein 4 | HGNC:3559 | 8q21.13 | GSE2956 | up |
| *EPB41L3* | erythrocyte membrane protein band 4.1 like 3 | HGNC:3380 | 18p11.31 | GSE2956 | up |
| *EP300* | E1A binding protein p300 | HGNC:3373 | 22q13.2 | GSE2956 | up |
| *ENAH* | ENAH actin regulator | HGNC:18271 | 1q42.12 | GSE2956 | up |
| *EMP1* | epithelial membrane protein 1 | HGNC:3333 | 12p13.1 | GSE2956 | up |
| *EIF5A* | eukaryotic translation initiation factor 5A | HGNC:3300 | 17p13.1 | GSE2956 | up |
| *EIF4E* | eukaryotic translation initiation factor 4E | HGNC:3287 | 4q23 | GSE2956 | up |
| *EIF3A* | eukaryotic translation initiation factor 3 subunit A | HGNC:3271 | 10q26.11 | GSE2956 | up |
| *EIF1AX* | eukaryotic translation initiation factor 1A X-linked | HGNC:3250 | Xp22.12 | GSE2956 | up |
| *DNAJB9* | DnaJ heat shock protein family (Hsp40) member B9 | HGNC:6968 | 7q31.1 | GSE2956 | up |
| *DMXL1* | Dmx like 1 | HGNC:2937 | 5q23.1 | GSE2956 | up |
| *DHRS2* | dehydrogenase/reductase 2 | HGNC:18349 | 14q11.2 | GSE2956 | up |
| *DDX18* | DEAD-box helicase 18 | HGNC:2741 | 2q14.1 | GSE2956 | up |
| *DCLRE1C* | DNA cross-link repair 1C | HGNC:17642 | 10p13 | GSE2956 | up |
| *DARS1* | aspartyl-tRNA synthetase 1 | HGNC:2678 | 2q21.3 | GSE2956 | up |
| *CCN1* | cellular communication network factor 1 | HGNC:2654 | 1p22.3 | GSE2956 | up |
| *CYP51A1* | cytochrome P450 family 51 subfamily A member 1 | HGNC:2649 | 7q21.2 | GSE2956 | up |
| *CYP1B1* | cytochrome P450 family 1 subfamily B member 1 | HGNC:2597 | 2p22.2 | GSE2956 | up |
| *CXCL14* | C-X-C motif chemokine ligand 14 | HGNC:10640 | 5q31.1 | GSE2956 | up |
| *CTSS* | cathepsin S | HGNC:2545 | 1q21.3 | GSE2956 | up |
| *CCN2* | cellular communication network factor 2 | HGNC:2500 | 6q23.2 | GSE2956 | up |
| *HAPLN1* | hyaluronan and proteoglycan link protein 1 | HGNC:2380 | 5q14.3 | GSE2956 | up |
| *EID1* | EP300 interacting inhibitor of differentiation 1 | HGNC:1191 | 15q21.1 | GSE2956 | up |
| *CREB1* | cAMP responsive element binding protein 1 | HGNC:2345 | 2q33.3 | GSE2956 | up |
| *CPM* | carboxypeptidase M | HGNC:2311 | 12q15 | GSE2956 | up |
| *COL1A1* | collagen type I alpha 1 chain | HGNC:2197 | 17q21.33 | GSE2956 | up |
| *CNN3* | calponin 3 | HGNC:2157 | 1p21.3 | GSE2956 | up |
| *CDC42EP4* | CDC42 effector protein 4 | HGNC:17147 | 17q25.1 | GSE2956 | up |
| *CDC42BPB* | CDC42 binding protein kinase beta | HGNC:1738 | 14q32.32 | GSE2956 | up |
| *CDC14A* | cell division cycle 14A | HGNC:1718 | 1p21.2 | GSE2956 | up |
| *CD36* | CD36 molecule | HGNC:1663 | 7q21.11 | GSE2956 | up |
| *CCNI* | cyclin I | HGNC:1595 | 4q21.1 | GSE2956 | up |
| *CAPZA2* | capping actin protein of muscle Z-line subunit alpha 2 | HGNC:1490 | 7q31.2 | GSE2956 | up |
| *CALR* | calreticulin | HGNC:1455 | 19p13.13 | GSE2956 | up |
| *CALM1* | calmodulin 1 | HGNC:1442 | 14q32.11 | GSE2956 | up |
| *CALD1* | caldesmon 1 | HGNC:1441 | 7q33 | GSE2956 | up |
| *C1QBP* | complement C1q binding protein | HGNC:1243 | 17p13.2 | GSE2956 | up |
| *BMP5* | bone morphogenetic protein 5 | HGNC:1072 | 6p12.1 | GSE2956 | up |
| *BAZ1A* | bromodomain adjacent to zinc finger domain 1A | HGNC:960 | 14q13.1-q13.2 | GSE2956 | up |
| *ATP6V0E1* | ATPase H+ transporting V0 subunit e1 | HGNC:863 | 5q35.1 | GSE2956 | up |
| *ATP5PO* | ATP synthase peripheral stalk subunit OSCP | HGNC:850 | 21q22.11 | GSE2956 | up |
| *ATP2B1* | ATPase plasma membrane Ca2+ transporting 1 | HGNC:814 | 12q21.33 | GSE2956 | up |
| *ARNT2* | aryl hydrocarbon receptor nuclear translocator 2 | HGNC:16876 | 15q25.1 | GSE2956 | up |
| *ARHGAP6* | Rho GTPase activating protein 6 | HGNC:676 | Xp22.2 | GSE2956 | up |
| *ANXA3* | annexin A3 | HGNC:541 | 4q21.21 | GSE2956 | up |
| *ANGPT2* | angiopoietin 2 | HGNC:485 | 8p23.1 | GSE2956 | up |
| *AMFR* | autocrine motility factor receptor | HGNC:463 | 16q13 | GSE2956 | up |
| *ALDH1A3* | aldehyde dehydrogenase 1 family member A3 | HGNC:409 | 15q26.3 | GSE2956 | up |
| *ADD3* | adducin 3 | HGNC:245 | 10q25.1-q25.2 | GSE2956 | up |
| *ADCY7* | adenylate cyclase 7 | HGNC:238 | 16q12.1 | GSE2956 | up |
| *ACTN1* | actinin alpha 1 | HGNC:163 | 14q24.1 | GSE2956 | up |
| *ACTG2* | actin gamma 2, smooth muscle | HGNC:145 | 2p13.1 | GSE2956 | up |
| *ACTB* | actin beta | HGNC:132 | 7p22.1 | GSE2956 | up |
| *ACTA2* | actin alpha 2, smooth muscle | HGNC:130 | 10q23.31 | GSE2956 | up |
| *ABHD3* | abhydrolase domain containing 3, phospholipase | HGNC:18718 | 18q11.2 | GSE2956 | up |
| *KIF26B* | kinesin family member 26B | HGNC:25484 | 1q44 | GSEShengjing | up |
| *ZNF596* | zinc finger protein 596 | HGNC:27268 | 8p23.3 | GSEShengjing | up |
| *TTPA* | alpha tocopherol transfer protein | HGNC:12404 | 8q12.3 | GSEShengjing | up |
| *TRIM52* | tripartite motif containing 52 | HGNC:19024 | 5q35.3 | GSEShengjing | up |
| *ARHGAP42* | Rho GTPase activating protein 42 | HGNC:26545 | 11q22.1 | GSEShengjing | up |
| *THSD7B* | thrombospondin type 1 domain containing 7B | HGNC:29348 | 2q22.1 | GSEShengjing | up |
| *TAS2R30* | taste 2 receptor member 30 | HGNC:19112 | 12p13.2 | GSEShengjing | up |
| *SPESP1* | sperm equatorial segment protein 1 | HGNC:15570 | 15q23 | GSEShengjing | up |
| *SOHLH2* | spermatogenesis and oogenesis specific basic helix-loop-helix 2 | HGNC:26026 | 13q13.3 | GSEShengjing | up |
| *SLITRK6* | SLIT and NTRK like family member 6 | HGNC:23503 | 13q31.1 | GSEShengjing | up |
| *SLC2A12* | solute carrier family 2 member 12 | HGNC:18067 | 6q23.2 | GSEShengjing | up |
| *PKP1* | plakophilin 1 | HGNC:9023 | 1q32.1 | GSEShengjing | up |
| *PCDHGB2* | protocadherin gamma subfamily B, 2 | HGNC:8709 | 5q31 | GSEShengjing | up |
| *PCDHGA2* | protocadherin gamma subfamily A, 2 | HGNC:8700 | 5q31.3 | GSEShengjing | up |
| *PARPBP* | PARP1 binding protein | HGNC:26074 | 12q23.2 | GSEShengjing | up |
| *OXCT1* | 3-oxoacid CoA-transferase 1 | HGNC:8527 | 5p13.1 | GSEShengjing | up |
| *MXRA5* | matrix remodeling associated 5 | HGNC:7539 | Xp22.33 | GSEShengjing | up |
| *MCOLN3* | mucolipin TRP cation channel 3 | HGNC:13358 | 1p22.3 | GSEShengjing | up |
| *MAP3K7CL* | MAP3K7 C-terminal like | HGNC:16457 | 21q21.3 | GSEShengjing | up |
| *MAP1LC3C* | microtubule associated protein 1 light chain 3 gamma | HGNC:13353 | 1q43 | GSEShengjing | up |
| *LPL* | lipoprotein lipase | HGNC:6677 | 8p21.3 | GSEShengjing | up |
| *LPAR6* | lysophosphatidic acid receptor 6 | HGNC:15520 | 13q14.2 | GSEShengjing | up |
| *KCNJ16* | potassium inwardly rectifying channel subfamily J member 16 | HGNC:6262 | 17q24.3 | GSEShengjing | up |
| *JRK* | Jrk helix-turn-helix protein | HGNC:6199 | 8q24.3 | GSEShengjing | up |
| *HRH1* | histamine receptor H1 | HGNC:5182 | 3p25.3 | GSEShengjing | up |
| *HLA-B* | major histocompatibility complex, class I, B | HGNC:4932 | 6p21.33 | GSEShengjing | up |
| *HCAR1* | hydroxycarboxylic acid receptor 1 | HGNC:4532 | 12q24.31 | GSEShengjing | up |
| *GRID1* | glutamate ionotropic receptor delta type subunit 1 | HGNC:4575 | 10q23.1-q23.2 | GSEShengjing | up |
| *GLRB* | glycine receptor beta | HGNC:4329 | 4q32.1 | GSEShengjing | up |
| *GABRB3* | gamma-aminobutyric acid type A receptor subunit beta3 | HGNC:4083 | 15q12 | GSEShengjing | up |
| *GABRA3* | gamma-aminobutyric acid type A receptor subunit alpha3 | HGNC:4077 | Xq28 | GSEShengjing | up |
| *FGF1* | fibroblast growth factor 1 | HGNC:3665 | 5q31.3 | GSEShengjing | up |
| *CENPB* | centromere protein B | HGNC:1852 | 20p13 | GSEShengjing | up |
| *CCL28* | C-C motif chemokine ligand 28 | HGNC:17700 | 5p12 | GSEShengjing | up |
| *BMP4* | bone morphogenetic protein 4 | HGNC:1071 | 14q22.2 | GSEShengjing | up |
| *AMIGO1* | adhesion molecule with Ig like domain 1 | HGNC:20824 | 1p13.3 | GSEShengjing | up |
| *ACCS* | 1-aminocyclopropane-1-carboxylate synthase homolog (inactive) | HGNC:23989 | 11p11.2 | GSEShengjing | up |
| *CFD* | complement factor D | HGNC:2771 | 19p13.3 | GSE19649 | down |
| *COL17A1* | collagen type XVII alpha 1 chain | HGNC:2194 | 10q25.1 | GSE19649 | down |
| *DKK1* | dickkopf WNT signaling pathway inhibitor 1 | HGNC:2891 | 10q21.1 | GSE19649 | down |
| *CXCL1* | C-X-C motif chemokine ligand 1 | HGNC:4602 | 4q13.3 | GSE19649 | down |
| *LAMA3* | laminin subunit alpha 3 | HGNC:6483 | 18q11.2 | GSE19649 | down |
| *PRUNE2* | prune homolog 2 with BCH domain | HGNC:25209 | 9q21.2 | GSE19649 | down |
| *F2R* | coagulation factor II thrombin receptor | HGNC:3537 | 5q13.3 | GSE19649 | down |
| *MOCOS* | molybdenum cofactor sulfurase | HGNC:18234 | 18q12.2 | GSE19649 | down |
| *SIK1* | salt inducible kinase 1 | HGNC:11142 | 21q22.3 | GSE19649 | down |
| *CHKB* | choline kinase beta | HGNC:1938 | 22q13.33 | GSE19649 | down |
| *SULF2* | sulfatase 2 | HGNC:20392 | 20q13.12 | GSE19649 | down |
| *CEMIP* | cell migration inducing hyaluronidase 1 | HGNC:29213 | 15q25.1 | GSE19649 | down |
| *TFRC* | transferrin receptor | HGNC:11763 | 3q29 | GSE19649 | down |
| *PAEP* | progestagen associated endometrial protein | HGNC:8573 | 9q34.3 | GSE19649 | down |
| *CTSK* | cathepsin K | HGNC:2536 | 1q21.3 | GSE19649 | down |
| *EMILIN2* | elastin microfibril interfacer 2 | HGNC:19881 | 18p11.32-p11.31 | GSE19649 | down |
| *SNCAIP* | synuclein alpha interacting protein | HGNC:11139 | 5q23.2 | GSE19649 | down |
| *IL2RB* | interleukin 2 receptor subunit beta | HGNC:6009 | 22q12.3 | GSE19649 | down |
| *C1S* | complement C1s | HGNC:1247 | 12p13.31 | GSE19649 | down |
| *HSPA1A* | heat shock protein family A (Hsp70) member 1A | HGNC:5232 | 6p21.33 | GSE19649 | down |
| *PAPLN* | papilin, proteoglycan like sulfated glycoprotein | HGNC:19262 | 14q24.2 | GSE19649 | down |
| *LEP* | leptin | HGNC:6553 | 7q32.1 | GSE19649 | down |
| *SERPINA3* | serpin family A member 3 | HGNC:16 | 14q32.13 | GSE19649 | down |
| *RBP1* | retinol binding protein 1 | HGNC:9919 | 3q23 | GSE19649 | down |
| *PNCK* | pregnancy up-regulated nonubiquitous CaM kinase | HGNC:13415 | Xq28 | GSE19649 | down |
| *OMD* | osteomodulin | HGNC:8134 | 9q22.31 | GSE19649 | down |
| *LYPD5* | LY6/PLAUR domain containing 5 | HGNC:26397 | 19q13.31 | GSE19649 | down |
| *ACKR1* | atypical chemokine receptor 1 (Duffy blood group) | HGNC:4035 | 1q23.2 | GSE19649 | down |
| *MUC20* | mucin 20, cell surface associated | HGNC:23282 | 3q29 | GSE19649 | down |
| *AOX1* | aldehyde oxidase 1 | HGNC:553 | 2q33.1 | GSE19649 | down |
| *STC1* | stanniocalcin 1 | HGNC:11373 | 8p21.2 | GSE19649 | down |
| *IL15* | interleukin 15 | HGNC:5977 | 4q31.21 | GSE19649 | down |
| *DHRS2* | dehydrogenase/reductase 2 | HGNC:18349 | 14q11.2 | GSE19649 | down |
| *MMP9* | matrix metallopeptidase 9 | HGNC:7176 | 20q13.12 | GSE19649 | down |
| *TMEM45A* | transmembrane protein 45A | HGNC:25480 | 3q12.2 | GSE19649 | down |
| *UPK1B* | uroplakin 1B | HGNC:12578 | 3q13.32 | GSE19649 | down |
| *OAF* | out at first homolog | HGNC:28752 | 11q23.3 | GSE19649 | down |
| *SYT8* | synaptotagmin 8 | HGNC:19264 | 11p15.5 | GSE19649 | down |
| *IL1B* | interleukin 1 beta | HGNC:5992 | 2q14.1 | GSE19649 | down |
| *FAM118A* | family with sequence similarity 118 member A | HGNC:1313 | 22q13.31 | GSE19649 | down |
| *HLA-G* | major histocompatibility complex, class I, G | HGNC:4964 | 6p22.1 | GSE19649 | down |
| *HSPB6* | heat shock protein family B (small) member 6 | HGNC:26511 | 19q13.12 | GSE19649 | down |
| *NR4A2* | nuclear receptor subfamily 4 group A member 2 | HGNC:7981 | 2q22-q23 | GSE19649 | down |
| *PRDM1* | PR/SET domain 1 | HGNC:9346 | 6q21 | GSE19649 | down |
| *SEMA5A* | semaphorin 5A | HGNC:10736 | 5p15.31 | GSE19649 | down |
| *RORB* | RAR related orphan receptor B | HGNC:10259 | 9q21.13 | GSE19649 | down |
| *TLE6* | TLE family member 6, subcortical maternal complex member | HGNC:30788 | 19p13.3 | GSE19649 | down |
| *CST6* | cystatin E/M | HGNC:2478 | 11q13.1 | GSE19649 | down |
| *HLA-DRB3* | major histocompatibility complex, class II, DR beta 3 | HGNC:4951 | 6p21.3 alternate reference locus | GSE19649 | down |
| *NDP* | norrin cystine knot growth factor NDP | HGNC:7678 | Xp11.3 | GSE19649 | down |
| *MGST1* | microsomal glutathione S-transferase 1 | HGNC:7061 | 12p12.3 | GSE19649 | down |
| *AHNAK2* | AHNAK nucleoprotein 2 | HGNC:20125 | 14q32.33 | GSE19649 | down |
| *DEFB1* | defensin beta 1 | HGNC:2766 | 8p23.1 | GSE19649 | down |
| *H2BC8* | H2B clustered histone 8 | HGNC:4746 | 6p22.2 | GSE19649 | down |
| *CILP* | cartilage intermediate layer protein | HGNC:1980 | 15q22.31 | GSE19649 | down |
| *ALDOC* | aldolase, fructose-bisphosphate C | HGNC:418 | 17q11.2 | GSE19649 | down |
| *NLGN4X* | neuroligin 4 X-linked | HGNC:14287 | Xp22.32-p22.31 | GSE19649 | down |
| *METTL27* | methyltransferase like 27 | HGNC:19068 | 7q11.23 | GSE19649 | down |
| *ABCC3* | ATP binding cassette subfamily C member 3 | HGNC:54 | 17q21.33 | GSE19649 | down |
| *SLC2A5* | solute carrier family 2 member 5 | HGNC:11010 | 1p36.23 | GSE19649 | down |
| *ANKRD37* | ankyrin repeat domain 37 | HGNC:29593 | 4q35.1 | GSE19649 | down |
| *WT1* | WT1 transcription factor | HGNC:12796 | 11p13 | GSE19649 | down |
| *IL1R2* | interleukin 1 receptor type 2 | HGNC:5994 | 2q11.2 | GSE19649 | down |
| *SPOCK1* | SPARC (osteonectin), cwcv and kazal like domains proteoglycan 1 | HGNC:11251 | 5q31.2 | GSE19649 | down |
| *C3* | complement C3 | HGNC:1318 | 19p13.3 | GSE19649 | down |
| *ISLR* | immunoglobulin superfamily containing leucine rich repeat | HGNC:6133 | 15q24.1 | GSE19649 | down |
| *SLPI* | secretory leukocyte peptidase inhibitor | HGNC:11092 | 20q13.12 | GSE19649 | down |
| *MT1G* | metallothionein 1G | HGNC:7399 | 16q13 | GSE19649 | down |
| *PCSK6* | proprotein convertase subtilisin/kexin type 6 | HGNC:8569 | 15q26.3 | GSE19649 | down |
| *IGFBP2* | insulin like growth factor binding protein 2 | HGNC:5471 | 2q35 | GSE19649 | down |
| *EPDR1* | ependymin related 1 | HGNC:17572 | 7p14.1 | GSE19649 | down |
| *RNFT2* | ring finger protein, transmembrane 2 | HGNC:25905 | 12q24.22 | GSE19649 | down |
| *HSD11B1* | hydroxysteroid 11-beta dehydrogenase 1 | HGNC:5208 | 1q32.2 | GSE19649 | down |
| *TMC4* | transmembrane channel like 4 | HGNC:22998 | 19q13.42 | GSE19649 | down |
| *HLA-DQA1* | major histocompatibility complex, class II, DQ alpha 1 | HGNC:4942 | 6p21.32 | GSE19649 | down |
| *HOXA5* | homeobox A5 | HGNC:5106 | 7p15.2 | GSE19649 | down |
| *GZMK* | granzyme K | HGNC:4711 | 5q11.2 | GSE19649 | down |
| *SCARA5* | scavenger receptor class A member 5 | HGNC:28701 | 8p21.1 | GSE19649 | down |
| *MOXD1* | monooxygenase DBH like 1 | HGNC:21063 | 6q23.2 | GSE19649 | down |
| *GZMA* | granzyme A | HGNC:4708 | 5q11.2 | GSE19649 | down |
| *KISS1R* | KISS1 receptor | HGNC:4510 | 19p13.3 | GSE19649 | down |
| *MLPH* | melanophilin | HGNC:29643 | 2q37.3 | GSE19649 | down |
| *ALDH1A2* | aldehyde dehydrogenase 1 family member A2 | HGNC:15472 | 15q21.3 | GSE19649 | down |
| *FOLR3* | folate receptor gamma | HGNC:3795 | 11q13.4 | GSE19649 | down |
| *MSLN* | mesothelin | HGNC:7371 | 16p13.3 | GSE19649 | down |
| *KIR2DL3* | killer cell immunoglobulin like receptor, two Ig domains and long cytoplasmic tail 3 | HGNC:6331 | 19q13.42 | GSE19649 | down |
| *BHLHE40* | basic helix-loop-helix family member e40 | HGNC:1046 | 3p26.1 | GSE19649 | down |
| *NKG7* | natural killer cell granule protein 7 | HGNC:7830 | 19q13.41 | GSE19649 | down |
| *KIR2DL4* | killer cell immunoglobulin like receptor, two Ig domains and long cytoplasmic tail 4 | HGNC:6332 | 19q13.42 | GSE19649 | down |
| *IGFBP6* | insulin like growth factor binding protein 6 | HGNC:5475 | 12q13.13 | GSE19649 | down |
| *SPRR2G* | small proline rich protein 2G | HGNC:11267 | 1q21.3 | GSE19649 | down |
| *EGFR* | epidermal growth factor receptor | HGNC:3236 | 7p11.2 | GSE19649 | down |
| *CYP4B1* | cytochrome P450 family 4 subfamily B member 1 | HGNC:2644 | 1p33 | GSE19649 | down |
| *AADAC* | arylacetamide deacetylase | HGNC:17 | 3q25.1 | GSE19649 | down |
| *CNR1* | cannabinoid receptor 1 | HGNC:2159 | 6q15 | GSE19649 | down |
| *SHISAL1* | shisa like 1 | HGNC:29335 | 22q13.31 | GSE19649 | down |
| *CD2* | CD2 molecule | HGNC:1639 | 1p13.1 | GSE19649 | down |
| *COX7B2* | cytochrome c oxidase subunit 7B2 | HGNC:24381 | 4p12 | GSE19649 | down |
| *GKN1* | gastrokine 1 | HGNC:23217 | 2p13.3 | GSE19649 | down |
| *CA12* | carbonic anhydrase 12 | HGNC:1371 | 15q22.2 | GSE19649 | down |
| *TFF3* | trefoil factor 3 | HGNC:11757 | 21q22.3 | GSE19649 | down |
| *GNLY* | granulysin | HGNC:4414 | 2p11.2 | GSE19649 | down |
| *BGN* | biglycan | HGNC:1044 | Xq28 | GSE19649 | down |
| *PCDH20* | protocadherin 20 | HGNC:14257 | 13q21.2 | GSE19649 | down |
| *SERPINA5* | serpin family A member 5 | HGNC:8723 | 14q32.13 | GSE19649 | down |
| *OR7D2* | olfactory receptor family 7 subfamily D member 2 | HGNC:8378 | 19p13.2 | GSE19649 | down |
| *JCHAIN* | joining chain of multimeric IgA and IgM | HGNC:5713 | 4q13.3 | GSE19649 | down |
| *SCGB2A1* | secretoglobin family 2A member 1 | HGNC:7051 | 11q12.3 | GSE19649 | down |
| *TTTY15* | testis-specific transcript, Y-linked 15 | HGNC:18567 | Yq11.221 | GSE2956 | down |
| *TSKS* | testis specific serine kinase substrate | HGNC:30719 | 19q13.33 | GSE2956 | down |
| *TPTE* | transmembrane phosphatase with tensin homology | HGNC:12023 | 21p11.2 | GSE2956 | down |
| *TNFRSF8* | TNF receptor superfamily member 8 | HGNC:11923 | 1p36.22 | GSE2956 | down |
| *TMPRSS3* | transmembrane serine protease 3 | HGNC:11877 | 21q22.3 | GSE2956 | down |
| *TIMP3* | TIMP metallopeptidase inhibitor 3 | HGNC:11822 | 22q12.3 | GSE2956 | down |
| *THRA* | thyroid hormone receptor alpha | HGNC:11796 | 17q21.1 | GSE2956 | down |
| *TAS2R16* | taste 2 receptor member 16 | HGNC:14921 | 7q31.32 | GSE2956 | down |
| *TAGLN2* | transgelin 2 | HGNC:11554 | 1q23.2 | GSE2956 | down |
| *SLC6A14* | solute carrier family 6 member 14 | HGNC:11047 | Xq23 | GSE2956 | down |
| *SLC3A1* | solute carrier family 3 member 1 | HGNC:11025 | 2p21 | GSE2956 | down |
| *SLC23A1* | solute carrier family 23 member 1 | HGNC:10974 | 5q31.2 | GSE2956 | down |
| *SLC23A2* | solute carrier family 23 member 2 | HGNC:10973 | 20p13 | GSE2956 | down |
| *SLCO4A1* | solute carrier organic anion transporter family member 4A1 | HGNC:10953 | 20q13.33 | GSE2956 | down |
| *SLC16A3* | solute carrier family 16 member 3 | HGNC:10924 | 17q25.3 | GSE2956 | down |
| *SIX6* | SIX homeobox 6 | HGNC:10892 | 14q23.1 | GSE2956 | down |
| *SIX2* | SIX homeobox 2 | HGNC:10888 | 2p21 | GSE2956 | down |
| *SIGLEC6* | sialic acid binding Ig like lectin 6 | HGNC:10875 | 19q13.41 | GSE2956 | down |
| *SRSF6* | serine and arginine rich splicing factor 6 | HGNC:10788 | 20q13.11 | GSE2956 | down |
| *SFRP1* | secreted frizzled related protein 1 | HGNC:10776 | 8p11.21 | GSE2956 | down |
| *SERPINB3* | serpin family B member 3 | HGNC:10569 | 18q21.33 | GSE2956 | down |
| *SDS* | serine dehydratase | HGNC:10691 | 12q24.13 | GSE2956 | down |
| *S100A4* | S100 calcium binding protein A4 | HGNC:10494 | 1q21.3 | GSE2956 | down |
| *RPS4Y1* | ribosomal protein S4 Y-linked 1 | HGNC:10425 | Yp11.2 | GSE2956 | down |
| *RORA* | RAR related orphan receptor A | HGNC:10258 | 15q22.2 | GSE2956 | down |
| *RNASE1* | ribonuclease A family member 1, pancreatic | HGNC:10044 | 14q11.2 | GSE2956 | down |
| *RASGRP1* | RAS guanyl releasing protein 1 | HGNC:9878 | 15q14 | GSE2956 | down |
| *RABL2A* | RAB, member of RAS oncogene family like 2A | HGNC:9799 | 2q14.1 | GSE2956 | down |
| *RAB9BP1* | RAB9B, member RAS oncogene family pseudogene 1 | HGNC:9793 | 5q21.2-q21.3 | GSE2956 | down |
| *RAB3B* | RAB3B, member RAS oncogene family | HGNC:9778 | 1p32.3 | GSE2956 | down |
| *QSOX1* | quiescin sulfhydryl oxidase 1 | HGNC:9756 | 1q25.2 | GSE2956 | down |
| *PVR* | PVR cell adhesion molecule | HGNC:9705 | 19q13.31 | GSE2956 | down |
| *TWF1* | twinfilin actin binding protein 1 | HGNC:9620 | 12q12 | GSE2956 | down |
| *PSCA* | prostate stem cell antigen | HGNC:9500 | 8q24.3 | GSE2956 | down |
| *PRL* | prolactin | HGNC:9445 | 6p22.3 | GSE2956 | down |
| *PPIE* | peptidylprolyl isomerase E | HGNC:9258 | 1p34.2 | GSE2956 | down |
| *POMZP3* | POM121 and ZP3 fusion | HGNC:9203 | 7q11.23 | GSE2956 | down |
| *PLAU* | plasminogen activator, urokinase | HGNC:9052 | 10q22.2 | GSE2956 | down |
| *PAPPA2* | pappalysin 2 | HGNC:14615 | 1q25.2 | GSE2956 | down |
| *PIK3CD* | phosphatidylinositol-4,5-bisphosphate 3-kinase catalytic subunit delta | HGNC:8977 | 1p36.22 | GSE2956 | down |
| *PGRMC1* | progesterone receptor membrane component 1 | HGNC:16090 | Xq24 | GSE2956 | down |
| *PAWR* | pro-apoptotic WT1 regulator | HGNC:8614 | 12q21.2 | GSE2956 | down |
| *REG3A* | regenerating family member 3 alpha | HGNC:8601 | 2p12 | GSE2956 | down |
| *PAK3* | p21 (RAC1) activated kinase 3 | HGNC:8592 | Xq23 | GSE2956 | down |
| *OR1E2* | olfactory receptor family 1 subfamily E member 2 | HGNC:8190 | 17p13.2 | GSE2956 | down |
| *OR12D3* | olfactory receptor family 12 subfamily D member 3 | HGNC:13963 | 6p22.1 | GSE2956 | down |
| *OR10C1* | olfactory receptor family 10 subfamily C member 1 | HGNC:8165 | 6p22.1 | GSE2956 | down |
| *NRF1* | nuclear respiratory factor 1 | HGNC:7996 | 7q32.2 | GSE2956 | down |
| *NR1D2* | nuclear receptor subfamily 1 group D member 2 | HGNC:7963 | 3p24.2 | GSE2956 | down |
| *NPY2R* | neuropeptide Y receptor Y2 | HGNC:7957 | 4q32.1 | GSE2956 | down |
| *NF1* | neurofibromin 1 | HGNC:7765 | 17q11.2 | GSE2956 | down |
| *MYH10* | myosin heavy chain 10 | HGNC:7568 | 17p13.1 | GSE2956 | down |
| *MYBPC1* | myosin binding protein C1 | HGNC:7549 | 12q23.2 | GSE2956 | down |
| *MX1* | MX dynamin like GTPase 1 | HGNC:7532 | 21q22.3 | GSE2956 | down |
| *MMP14* | matrix metallopeptidase 14 | HGNC:7160 | 14q11.2 | GSE2956 | down |
| *MIF* | macrophage migration inhibitory factor | HGNC:7097 | 22q11.23 | GSE2956 | down |
| *CD99* | CD99 molecule (Xg blood group) | HGNC:7082 | Xp22.32 and Yp11.3 | GSE2956 | down |
| *SMAD5* | SMAD family member 5 | HGNC:6771 | 5q31.1 | GSE2956 | down |
| *LPL* | lipoprotein lipase | HGNC:6677 | 8p21.3 | GSE2956 | down |
| *LHB* | luteinizing hormone subunit beta | HGNC:6584 | 19q13.33 | GSE2956 | down |
| *LAMP1* | lysosomal associated membrane protein 1 | HGNC:6499 | 13q34 | GSE2956 | down |
| *KRT6B* | keratin 6B | HGNC:6444 | 12q13.13 | GSE2956 | down |
| *KRT6A* | keratin 6A | HGNC:6443 | 12q13.13 | GSE2956 | down |
| *TTLL5* | tubulin tyrosine ligase like 5 | HGNC:19963 | 14q24.3 | GSE2956 | down |
| *ITIH2* | inter-alpha-trypsin inhibitor heavy chain 2 | HGNC:6167 | 10p14 | GSE2956 | down |
| *ITGB3* | integrin subunit beta 3 | HGNC:6156 | 17q21.32 | GSE2956 | down |
| *IRF9* | interferon regulatory factor 9 | HGNC:6131 | 14q12 | GSE2956 | down |
| *ISG15* | ISG15 ubiquitin like modifier | HGNC:4053 | 1p36.33 | GSE2956 | down |
| *CADM1* | cell adhesion molecule 1 | HGNC:5951 | 11q23.3 | GSE2956 | down |
| *IGFBP1* | insulin like growth factor binding protein 1 | HGNC:5469 | 7p12.3 | GSE2956 | down |
| *IGF1* | insulin like growth factor 1 | HGNC:5464 | 12q23.2 | GSE2956 | down |
| *IFNG* | interferon gamma | HGNC:5438 | 12q15 | GSE2956 | down |
| *IFI30* | IFI30 lysosomal thiol reductase | HGNC:5398 | 19p13.11 | GSE2956 | down |
| *HES1* | hes family bHLH transcription factor 1 | HGNC:5192 | 3q29 | GSE2956 | down |
| *HPSE* | heparanase | HGNC:5164 | 4q21.23 | GSE2956 | down |
| *HNRNPR* | heterogeneous nuclear ribonucleoprotein R | HGNC:5047 | 1p36.12 | GSE2956 | down |
| *HADHA* | hydroxyacyl-CoA dehydrogenase trifunctional multienzyme complex subunit alpha | HGNC:4801 | 2p23.3 | GSE2956 | down |
| *H2BC9* | H2B clustered histone 9 | HGNC:4755 | 6p22.2 | GSE2956 | down |
| *H2AC18* | H2A clustered histone 18 | HGNC:4736 | 1q21.2 | GSE2956 | down |
| *GLRA1* | glycine receptor alpha 1 | HGNC:4326 | 5q33.1 | GSE2956 | down |
| *GGT1* | gamma-glutamyltransferase 1 | HGNC:4250 | 22q11.23 | GSE2956 | down |
| *GFRA1* | GDNF family receptor alpha 1 | HGNC:4243 | 10q25.3 | GSE2956 | down |
| *FXYD6* | FXYD domain containing ion transport regulator 6 | HGNC:4030 | 11q23.3 | GSE2956 | down |
| *FSTL3* | follistatin like 3 | HGNC:3973 | 19p13.3 | GSE2956 | down |
| *FST* | follistatin | HGNC:3971 | 5q11.2 | GSE2956 | down |
| *FRZB* | frizzled related protein | HGNC:3959 | 2q32.1 | GSE2956 | down |
| *FOS* | Fos proto-oncogene, AP-1 transcription factor subunit | HGNC:3796 | 14q24.3 | GSE2956 | down |
| *FN1* | fibronectin 1 | HGNC:3778 | 2q35 | GSE2956 | down |
| *KDM2A* | lysine demethylase 2A | HGNC:13606 | 11q13.2 | GSE2956 | down |
| *F11* | coagulation factor XI | HGNC:3529 | 4q35.2 | GSE2956 | down |
| *ESRRG* | estrogen related receptor gamma | HGNC:3474 | 1q41 | GSE2956 | down |
| *EPHA5* | EPH receptor A5 | HGNC:3389 | 4q13.1-q13.2 | GSE2956 | down |
| *EMD* | emerin | HGNC:3331 | Xq28 | GSE2956 | down |
| *EIF1AY* | eukaryotic translation initiation factor 1A Y-linked | HGNC:3252 | Yq11.223 | GSE2956 | down |
| *EGR1* | early growth response 1 | HGNC:3238 | 5q31.2 | GSE2956 | down |
| *EGLN3* | egl-9 family hypoxia inducible factor 3 | HGNC:14661 | 14q13.1 | GSE2956 | down |
| *EFNB2* | ephrin B2 | HGNC:3227 | 13q33.3 | GSE2956 | down |
| *DUSP3* | dual specificity phosphatase 3 | HGNC:3069 | 17q21.31 | GSE2956 | down |
| *EPYC* | epiphycan | HGNC:3053 | 12q21.33 | GSE2956 | down |
| *DNAJC3* | DnaJ heat shock protein family (Hsp40) member C3 | HGNC:9439 | 13q32.1 | GSE2956 | down |
| *DNAH17* | dynein axonemal heavy chain 17 | HGNC:2946 | 17q25.3 | GSE2956 | down |
| *DHX9* | DExH-box helicase 9 | HGNC:2750 | 1q25.3 | GSE2956 | down |
| *DDX17* | DEAD-box helicase 17 | HGNC:2740 | 22q13.1 | GSE2956 | down |
| *DDX3Y* | DEAD-box helicase 3 Y-linked | HGNC:2699 | Yq11.221 | GSE2956 | down |
| *CYP1A1* | cytochrome P450 family 1 subfamily A member 1 | HGNC:2595 | 15q24.1 | GSE2956 | down |
| *CYBB* | cytochrome b-245 beta chain | HGNC:2578 | Xp21.1-p11.4 | GSE2956 | down |
| *CTSZ* | cathepsin Z | HGNC:2547 | 20q13.32 | GSE2956 | down |
| *CRYM* | crystallin mu | HGNC:2418 | 16p12.2 | GSE2956 | down |
| *CRIP1* | cysteine rich protein 1 | HGNC:2360 | 14q32.33 | GSE2956 | down |
| *CRH* | corticotropin releasing hormone | HGNC:2355 | 8q13.1 | GSE2956 | down |
| *COL4A3* | collagen type IV alpha 3 chain | HGNC:2204 | 2q36.3 | GSE2956 | down |
| *CMAHP* | cytidine monophospho-N-acetylneuraminic acid hydroxylase, pseudogene | HGNC:2098 | 6p22.3 | GSE2956 | down |
| *CLU* | clusterin | HGNC:2095 | 8p21.1 | GSE2956 | down |
| *CLTB* | clathrin light chain B | HGNC:2091 | 5q35.2 | GSE2956 | down |
| *CLIC3* | chloride intracellular channel 3 | HGNC:2064 | 9q34.3 | GSE2956 | down |
| *CLCA2* | chloride channel accessory 2 | HGNC:2016 | 1p22.3 | GSE2956 | down |
| *CGB5* | chorionic gonadotropin subunit beta 5 | HGNC:16452 | 19q13.33 | GSE2956 | down |
| *CEACAM4* | CEA cell adhesion molecule 4 | HGNC:1816 | 19q13.2 | GSE2956 | down |
| *CDH11* | cadherin 11 | HGNC:1750 | 16q21 | GSE2956 | down |
| *CDC27* | cell division cycle 27 | HGNC:1728 | 17q21.32 | GSE2956 | down |
| *CD14* | CD14 molecule | HGNC:1628 | 5q31.3 | GSE2956 | down |
| *CAPN3* | calpain 3 | HGNC:1480 | 15q15.1 | GSE2956 | down |
| *FAM120A* | family with sequence similarity 120A | HGNC:13247 | 9q22.31 | GSE2956 | down |
| *B3GNT4* | UDP-GlcNAc:betaGal beta-1,3-N-acetylglucosaminyltransferase 4 | HGNC:15683 | 12q24.31 | GSE2956 | down |
| *ATP5MC2* | ATP synthase membrane subunit c locus 2 | HGNC:842 | 12q13.13 | GSE2956 | down |
| *ATP2B4* | ATPase plasma membrane Ca2+ transporting 4 | HGNC:817 | 1q32.1 | GSE2956 | down |
| *AREG* | amphiregulin | HGNC:651 | 4q13.3 | GSE2956 | down |
| *AQP3* | aquaporin 3 (Gill blood group) | HGNC:636 | 9p13.3 | GSE2956 | down |
| *ALPP* | alkaline phosphatase, placental | HGNC:439 | 2q37.1 | GSE2956 | down |
| *AGPAT2* | 1-acylglycerol-3-phosphate O-acyltransferase 2 | HGNC:325 | 9q34.3 | GSE2956 | down |
| *ADAM28* | ADAM metallopeptidase domain 28 | HGNC:206 | 8p21.2 | GSE2956 | down |
| *ABCG4* | ATP binding cassette subfamily G member 4 | HGNC:13884 | 11q23.3 | GSE2956 | down |
| *ABCG1* | ATP binding cassette subfamily G member 1 | HGNC:73 | 21q22.3 | GSE2956 | down |
| *CXCR1* | C-X-C motif chemokine receptor 1 | HGNC:6026 | 2q35 | GSEShengjing | down |
| *ZFP2* | ZFP2 zinc finger protein | HGNC:26138 | 5q35.3 | GSEShengjing | down |
| *TSPAN14* | tetraspanin 14 | HGNC:23303 | 10q23.1 | GSEShengjing | down |
| *TMPRSS2* | transmembrane serine protease 2 | HGNC:11876 | 21q22.3 | GSEShengjing | down |
| *TMEM79* | transmembrane protein 79 | HGNC:28196 | 1q22 | GSEShengjing | down |
| *TMEM74B* | transmembrane protein 74B | HGNC:15893 | 20p13 | GSEShengjing | down |
| *TMCC2* | transmembrane and coiled-coil domain family 2 | HGNC:24239 | 1q32.1 | GSEShengjing | down |
| *SLC4A1* | solute carrier family 4 member 1 (Diego blood group) | HGNC:11027 | 17q21.31 | GSEShengjing | down |
| *SLC28A1* | solute carrier family 28 member 1 | HGNC:11001 | 15q25.3 | GSEShengjing | down |
| *RGL2* | ral guanine nucleotide dissociation stimulator like 2 | HGNC:9769 | 6p21.32 | GSEShengjing | down |
| *OVCH2* | ovochymase 2 | HGNC:29970 | 11p15.4 | GSEShengjing | down |
| *NUDT4* | nudix hydrolase 4 | HGNC:8051 | 12q22 | GSEShengjing | down |
| *NTRK2* | neurotrophic receptor tyrosine kinase 2 | HGNC:8032 | 9q21.33 | GSEShengjing | down |
| *NLRP10* | NLR family pyrin domain containing 10 | HGNC:21464 | 11p15.4 | GSEShengjing | down |
| *MEFV* | MEFV innate immuity regulator, pyrin | HGNC:6998 | 16p13.3 | GSEShengjing | down |
| *LEPROTL1* | leptin receptor overlapping transcript like 1 | HGNC:6555 | 8p12 | GSEShengjing | down |
| *KRT15* | keratin 15 | HGNC:6421 | 17q21.2 | GSEShengjing | down |
| *ISY1-RAB43* | ISY1-RAB43 readthrough | HGNC:42969 | 3q21.3 | GSEShengjing | down |
| *IFIT1B* | interferon induced protein with tetratricopeptide repeats 1B | HGNC:23442 | 10q23.31 | GSEShengjing | down |
| *HLA-B* | major histocompatibility complex, class I, B | HGNC:4932 | 6p21.33 | GSEShengjing | down |
| *HES4* | hes family bHLH transcription factor 4 | HGNC:24149 | 1p36.33 | GSEShengjing | down |
| *HBM* | hemoglobin subunit mu | HGNC:4826 | 16p13.3 | GSEShengjing | down |
| *GPIHBP1* | glycosylphosphatidylinositol anchored high density lipoprotein binding protein 1 | HGNC:24945 | 8q24.3 | GSEShengjing | down |
| *GPER1* | G protein-coupled estrogen receptor 1 | HGNC:4485 | 7p22.3 | GSEShengjing | down |
| *FCN3* | ficolin 3 | HGNC:3625 | 1p36.11 | GSEShengjing | down |
| *FCN1* | ficolin 1 | HGNC:3623 | 9q34.3 | GSEShengjing | down |
| *TENT5C* | terminal nucleotidyltransferase 5C | HGNC:24712 | 1p12 | GSEShengjing | down |
| *EPB42* | erythrocyte membrane protein band 4.2 | HGNC:3381 | 15q15.2 | GSEShengjing | down |
| *ELOVL4* | ELOVL fatty acid elongase 4 | HGNC:14415 | 6q14.1 | GSEShengjing | down |
| *DAG1* | dystroglycan 1 | HGNC:2666 | 3p21.31 | GSEShengjing | down |
| *CXCR2* | C-X-C motif chemokine receptor 2 | HGNC:6027 | 2q35 | GSEShengjing | down |
| *CPE* | carboxypeptidase E | HGNC:2303 | 4q32.3 | GSEShengjing | down |
| *CMTM1* | CKLF like MARVEL transmembrane domain containing 1 | HGNC:19172 | 16q21 | GSEShengjing | down |
| *CLDN12* | claudin 12 | HGNC:2034 | 7q21.13 | GSEShengjing | down |
| *CDKN1A* | cyclin dependent kinase inhibitor 1A | HGNC:1784 | 6p21.2 | GSEShengjing | down |
| *CA4* | carbonic anhydrase 4 | HGNC:1375 | 17q23.1 | GSEShengjing | down |
| *BOK* | BCL2 family apoptosis regulator BOK | HGNC:1087 | 2q37.3 | GSEShengjing | down |
| *ANK1* | ankyrin 1 | HGNC:492 | 8p11.21 | GSEShengjing | down |
| *ALAS2* | 5'-aminolevulinate synthase 2 | HGNC:397 | Xp11.21 | GSEShengjing | down |
| *ADGRE2* | adhesion G protein-coupled receptor E2 | HGNC:3337 | 19p13.12 | GSEShengjing | down |
| *ABCF1* | ATP binding cassette subfamily F member 1 | HGNC:70 | 6p21.33 | GSEShengjing | down |
| *ABCA12* | ATP binding cassette subfamily A member 12 | HGNC:14637 | 2q35 | GSEShengjing | down |
