# Supplementary material for: Biological networks in gestational diabetes mellitus: insights into the mechanism of crosstalk between long non-coding RNA and N6-methyladenine modification
Source: BMC Pregnancy Childbirth. 2022 May 3;22:384. doi: 10.1186/s12884-022-04716-w (PMC9066898; doi:10.1186/s12884-022-04716-w)
Supplement: Supplementary file 3 — Additional file 3: Table S3. Clinical characteristics of patients included in the study. [file 12884_2022_4716_MOESM3_ESM.docx]

**Table S3. Clinical characteristics of patients included in the study**

| **Parameter** | **GDM**  **(n = 32)** | **NGT**  **(n = 32)** | ***P* value** |
| --- | --- | --- | --- |
| **Maternal age (years)** | 33.50 ± 5.15 | 31.28 ± 4.51 | 0.072 |
| **Pre-pregnancy BMI (kg/m^2^)** | 24.61 ± 5.07 | 26.08 ±5.81 | 0.285 |
| **Weight gain during pregnancy (kg)** | 12.28 ± 2.70 | 9.47 ± 2.95 | 0.000** |
| **Gravidity history** | 2.25 ± 1.22 | 1.91 ±1.32 | 0.285 |
| **Parity history** | 0.41 ± 0.56 | 0.34 ± 0.48 | 0.634 |
| **Gestational age (days)** | 269.16 ± 7.03 | 273.16 ± 7.03 | 0.061 |
| **OGTT at diagnosis of GDM or at 24~28 weeks of gestation** |  |  |  |
| **0 min glucose (mmol/L)** | 5.27 ± 0.55 | 4.55 ± 0.22 | 0.000** |
| **60 min glucose (mmol/L)** | 9.94 ± 1.86 | 7.00 ± 1.17 | 0.000** |
| **120 min glucose (mmol/L)** | 8.70 ± 1.74 | 6.26 ± 0.93 | 0.000** |
| **FPG at delivery (mmol/L)** | 4.80 ± 0.56 | 4.30 ± 0.51 | 0.001** |
| **HbA1c at delivery (%)** | 5.55 ± 0.52 | 5.30 ± 0.33 | 0.025* |
| **Baby's sex (female) (n[n%])** | 17 (53.13) | 17 (53.13) | 1.000 |
| **Birth weight (g)** | 3452.50 ± 442.91 | 3342.97 ± 482.33 | 0.347 |
| **Apgar score (1 min)** | 9.91 ± 0.53 | 9.91 ± 0.39 | 1.000 |

**Abbreviations**

GDM: gestational diabetes mellitus; BMI: body mass index; NGT: normal glucose tolerane; OGTT: oral glucose tolerance test; FPG: fasting blood glucose; HbA1c: glycosylated hemoglobin.

Note: Data are expressed as mean ± standard deviation or n (n%), * *P* < 0.05, ** *P* < 0.01.
